# Don’t Shut the Stable Door after the Phage Has Bolted—The Importance of Bacteriophage Inactivation in Food Environments

**DOI:** 10.3390/v11050468

**Published:** 2019-05-22

**Authors:** Julia Sommer, Christoph Trautner, Anna Kristina Witte, Susanne Fister, Dagmar Schoder, Peter Rossmanith, Patrick-Julian Mester

**Affiliations:** 1 Christian Doppler Laboratory for Monitoring of Microbial Contaminants, Department for Farm Animal and Public Health in Veterinary Medicine, University of Veterinary Medicine, Veterinaerplatz 1, 1210 Vienna, Austria; julia.sommer@vetmeduni.ac.at (J.S.); christoph.trautner@vetmeduni.ac.at (C.T.); anna.witte@vetmeduni.ac.at (A.K.W.); peter.rossmanith@vetmeduni.ac.at (P.R.); 2 Unit of Food Microbiology, Institute of Food Safety, Food Technology and Veterinary Public Health, Department for Farm Animal and Public Health in Veterinary Medicine, University of Veterinary Medicine, Veterinaerplatz 1, 1210 Vienna, Austria; dagmar.schoder@vetmeduni.ac.at; 3 HTK Hygiene Technologie Kompetenzzentrum GmbH, Buger Str. 80, 96049 Bamberg, Germany; 4 Former member of Christian Doppler Laboratory for Monitoring of Microbial Contaminants, Institute of Milk Hygiene, Milk Technology and Food Science, Department for Farm Animal and Public Veterinary Health, University of Veterinary Medicine, Veterinärplatz 1, 1210 Vienna, Austria; susanne.fister1@gmail.com

**Keywords:** bacteriophage, commercially available phages, virus, disinfectant, food industry, antiviral strategies, disinfectant strategies

## Abstract

In recent years, a new potential measure against foodborne pathogenic bacteria was rediscovered—bacteriophages. However, despite all their advantages, in connection to their widespread application in the food industry, negative consequences such as an uncontrolled phage spread as well as a development of phage resistant bacteria can occur. These problems are mostly a result of long-term persistence of phages in the food production environment. As this topic has been neglected so far, this article reviews the current knowledge regarding the effectiveness of disinfectant strategies for phage inactivation and removal. For this purpose, the main commercial phage products, as well as their application fields are first discussed in terms of applicable inactivation strategies and legal regulations. Secondly, an overview of the effectiveness of disinfectants for bacteriophage inactivation in general and commercial phages in particular is given. Finally, this review outlines a possible strategy for users of commercial phage products in order to improve the effectiveness of phage inactivation and removal after application.

## 1. Introduction—Advantages and Disadvantages of Commercially Available Phage Products Used in Food Environments

**Figure 1:**
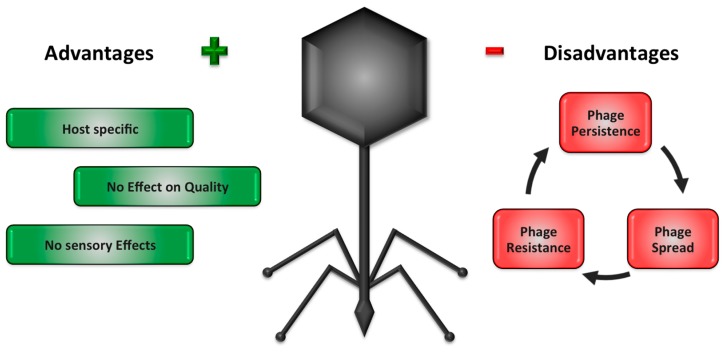
Advantages and disadvantages of commercially available phage products used in food environments.

Virus transmitted diseases are and always have been a constant challenge to humanity. Due to the increased mobility of people, animals and goods on a global scale, the spread of viral outbreaks is easier than ever [1,2,3], and especially foodborne viruses as well as viral zoonosis have come to the fore in recent years [4,5,6,7]. In addition to foodborne viruses, other classes of viruses, bacteriophages, are also highly relevant for the food industry as they can disrupt commercially important fermentative processes, for instance, in the dairy and soybean-based fermented food industry [8,9,10,11]. As adequate measures to prevent bacteriophage (hereafter phage) contamination or inactivate them are limited [12,13], phages have been considered as enemies in the food industry as well as for the medical sector for a long time. However, nowadays the attitude towards them has changed and phages have gained more interest as a weapon against pathogenic microorganisms [14,15]. For bacteria ranging from *Escherichia coli* [16,17,18], *Listeria monocytogenes* [19,20] to different *Salmonella* strains [21,22,23], commercial phage products are available now [24]. So far, many studies investigated the positive effects of phages against their target bacteria in food matrices and thus strengthening their reputation and their authority as an alternative to traditional disinfectants [25,26]. The main advantages of phages in comparison to commonly used chemical disinfectants and physical disinfectant measures, are their host-specificity and their negligible impact on sensory and quality characteristics of food (Figure 1) [9,24,27]. As other reviews such as O´Sullivan et al. [9], Moye et al. [24] and Sillankorva et al. [27] have already described the advantages of phages in agriculture, food production and food processing environments, this review will not discuss those advantages in detail.

Unfortunately, as it is the case with most routinely used disinfectant applications, there is a rising number of reports regarding negative aspects connected to phage application in the food industry [28,29,30]. Recently, our working group described two major problems with the routine use of phages in the food industry [31]: The development of phage-resistant bacteria as well as uncontrolled phage spread from the point of care (POC), which is a serious threat for subsequent diagnostic methods and can threaten routine monitoring. Loc-Carrillo et al. [28] discussed the importance of the correct choice of phages and the usage of phage cocktails to guarantee the highest possible virulence and avoid problems such as phage persistence. According to the authors, the phage of choice has to be a lytic phage, as temperate phages lead to an increased transfer of virulence factors between bacterial cells [28]. These conclusions have also been described by Endersen et al. [29]. Furthermore, the article of Loc-Carrillo mentioned the possible interactions with the human immune system (especially when phages used as a pharmaceutical drug), as the lysis of bacteria can lead to a release of bacterial toxins and protein-fragments [28]. Teng-Hern et al. [30] described the arise of phage-resistant mutants and possible routes for resistance development. Further, they discussed an interaction with the immune system, where the phage count could be substantially lowered via an immune response reaction. However, other factors can also crucially minimize the phage concentration, such as unspecific binding on food matrices or reduction due to gastric stress by oral application [30]. It has been previously reported that the development of phage-resistant mutants is almost impossible to completely prevent, especially in difficult to clean and disinfect niches, which are present in the food processing environment [31,32,33,34,35]. Some studies already indicated that particularly the used multiplicity of infection (MOI) is an essential parameter in the development of phage-resistant mutants [36,37,38]. Hosseinidoust et al. [36] concluded that a combined use with antibiotics or disinfectants could be a promising strategy to minimize the risk of development of phage-resistant mutants. As a result, different studies have already investigated the influence of antibiotics and disinfectants on the bacterial-phage-system, whereas synergistic effects of antibiotics [39,40] as well as antagonistic effects of disinfectants [41] on this ecosystem were observed. More often, however, the use of phage mixtures and rotations are discussed to avoid or at least limit the development of resistant mutants [30,42].

In addition to the development of phage-resistant mutants, the phage spread can also lead to serious problems. One main problem associated with the phage spread is the increasing risk of false negative results in bacterial growth-based routine detection of bacterial pathogens [31,43,44]. These problems are often not limited to the facility where the phages are inserted, as phages directly applied on food can be easily transferred from one facility to another [31]. An enhanced potential of the passive phage spread is strongly correlated to the recommended application manual, whereby the majority of commercially available phage products are suggested as spray use (see Table S1) [16,19,22,45]. In comparison to the primary user of such phage products, subsequent food processors are disadvantaged, as it is actually impossible to know if phages have been spread in the secondary facility [31].

The easiest strategy to limit the development of these problems is to efficiently limit the high persistence of phages in food processing plants. Unfortunately, while commercially available phage products for biocontrol have become widespread during the last 15 years, disinfectant strategies for the inactivation of those have been neglected. One of the reasons was probably the long-standing opinion that phages are completely harmless and would ultimately “disappear after killing the last bacterium” [46]. However, recent studies have shown that this is not the case and phages can persist in the agriculture and food environment for at least four months [47,48,49]. For example, Fister et al. [48] could show that phage P100 (commercially available as PhageGuard Listex™) can persist over 120 days in smear water at 4 °C and 10 °C, while Allué-Guardia et al. [50] have shown that different temperatures and pH-values had almost no impact on the induced phage titre after one month.

The persistence of phages can be traced back to their high physicochemical stability against environmental influences, such as pH, temperature, salinity, UV-light or commonly used disinfectants [47,48,51,52,53,54,55,56,57,58]. In general, most phages are stable at high salt concentrations up to 4.5 M NaCl [48,59] as well as in the pH range between pH 4 to pH 10 [48,50,59,60,61,62], however there are notable exceptions and for instance the phage P100 was reported to withstand one hour of incubation at ≤pH 2 and ≥12 [48]. A similar variation has been reported also in terms of the thermal stability of phages. While some phages can show a moderate thermal stability and withstand up to 62 °C for 20 min [59,62,63], for example the coliphage HY01 was only reduced by 3 log_10_ units after 12 h of incubation at 60 °C [62]. Such high thermal resistance was also reported in the study of Jurczak-Kurek et al. [59], where the authors investigated 83 phages in terms of their stability to various stresses. The authors found a highly variable stability range even for phages from the same order, with one phage being able to withstand incubation at 95 °C, and concluded that the stability range has to be determined for each phage individually [59]. Similar findings have been previously reported for economically important lactic acid bacteria (LAB) phages and where the need for proper and thorough inactivation and disinfection strategies has been clearly stated [12,59,64,65,66,67].

The lack of efficient inactivation or removal of phages in the food environment can end up in a “never ending cycle”, whereas the indirect phage spread leads again to persistence of phages and to an increased formation of niches, where phage-resistant mutants can develop [31]. For prevention of these and similar problems a concrete declaration of applied phages, as well as adequate inactivation and removal measures are desperately needed. Regarding one of these important points, the inactivation, also described in this review as disinfection of commercially available phages can be summed up by one easy question: How do I get rid of them after application?

In order to be able to answer this question, this review will tackle three points. First of all, “Who is there?” meaning which commercial phage products are available and for which field of application and application form. Secondly, “What can I do?” meaning what antiviral strategies can be undertaken in respect to phage application but also regarding the limitations of approved disinfectants for the food and feed industry? And finally, yet importantly, “Would it be working?” meaning if food manufacturers can readily trust their disinfectant strategy to be working against their phage product.

## 2. Commercial Phage Products, Their Application Field and Legal Framework

While the application of phages for medical purposes has a long tradition in different parts of the world [68,69,70], in the food sector phages have been commercially available as biological disinfectants against various foodborne pathogens as well as food spoilage bacteria, for about two decades [9,24,71,72,73,74,75,76,77,78,79]. Among their many advantages, the use of phages as biological disinfectants is highly advantageous compared to physical or chemical disinfectant measures due to their limited to none impact on sensory or quality parameters of the processed food [77,80,81]. Due to such direct-on-food intervention measures of phages, there are supposed to be stringent regulations by the Food and Drug Administration (FDA), the European Food Safety Authority (EFSA) and other agencies, which qualify them for safe usage [22,82,83,84,85]. However, in reality phages are not only applied for direct-on-food intervention measures. This creates, together with the lack of complete disclosure requirements of individual ingredients and because of various loopholes such as the protection of trademark claims, a certain legislative “Grey Zone” [86,87,88]. For example, in commercially available phage cocktails, it is not necessary to fully disclose all included phages individually (see Table S2), while for a patent full disclosure is needed (see Table S3) [89]. Phages are usually not legally regulated as a food additive but can also be used as a processing aid, a bio-pesticide or a drug, depending on their field of application [86,87,89,90,91]. Unlike a food additive, for example, a processing aid need not be declared on the product, as these substances are not consumed as a food by itself. In addition, the legal regulation further claims that if the presence of residue of a substance is (i) unintentional but technically unavoidable, (ii) not harmful, as well as iii) does not have any technological effects on the final product, a declaration as a processing aid will be legit [87,90]. As this review is dealing with the issue of “How to remove phages after successful application?”, from our point of view it makes sense to first look at the different application fields of commercial phage products, disregarding their legal regulation term, as for the problems resulting from their unwanted persistence their labelling makes no difference.

**Figure 2:**
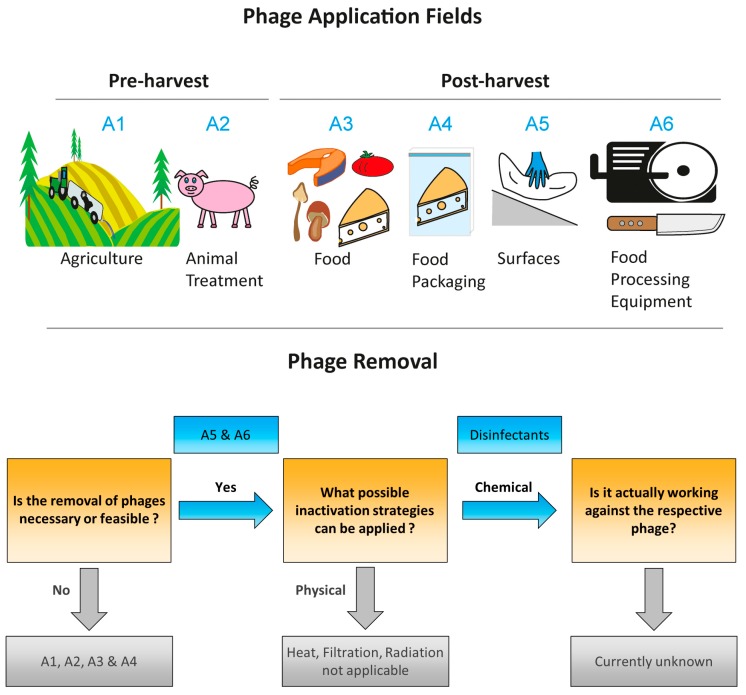
Fields of applications and consideration for adequate phage removal.

In Table 1, the current commercially available phage products, including their taxonomy, their respective bacterial target and their intended field of application are listed [16,17,18,19,20,21,22,23,24]. With respect to the topic of this review, the different stages in the food chain where the phages are intended to be applied are separated into a pre-harvest as well as a post-harvest environment (Figure 2) [24,42,92,93].

The pre-harvest application field includes the usage of phages in agriculture against plant specific pathogens (Figure 2(A1)) [94,95,96], for treatment of animals prior to slaughter [97] and as a direct animal treatment applied over the feed to ensure the physical health of the animal (Figure 2(A2)) [98]. Given the nature of such pre-harvest application fields, immediate phage removal after application is undesired and especially in the case of agriculture neither economic nor ecological, given the spacious treated area [92,99,100]. The same is of course true if living animals are directly treated with phages [98,101,102,103]. An exception would be the case of stables for animal rearing [104] where niches for phage persistence can occur and the change of different rearing groups would allow appropriate inactivation measures, however this is more related to surface disinfection which is discussed later on. In principle, for pre-harvest application fields, an efficient removal of phage products after application in order to prevent a phage spread and the development of phage-resistant mutants is quite unfeasible and these problems have to be approached with strategies such as phage-rotations and consistent monitoring [8,9,28]. Consequently, the limitation of phage persistence in niches and biofilms through effective disinfectant measures is best applied in the post-harvest application fields [15,105,106,107,108].

The post-harvest application field of commercial phages can best be divided into direct-on food application, food packaging, surfaces and food processing equipment (Figure 2(A3–A6)). As for the pre-harvest applications, for direct-on food application and food packaging, the phage inactivation after application is undesired or economically not feasible. In both of those applications, the goal is to minimize the growth of pathogens and spoilage organisms during storage or retail and thus, an inactivation subsequent to their application would be counterproductive and thus again, the potential problem associated with the phage usage has to be tackled with different solutions such as phage-rotations and consistent monitoring.

So overall, that leaves the treatment of surfaces (A5) and food processing equipment (A6) as the most promising intervention point, where the persistence of phages can be effectively approached. Given the nature of these two application fields, the main disinfection measures are chemical antimicrobials, as physical disinfection strategies are only applicable to very limited scenarios [77,80,81]. While chemical antimicrobials are already applied in the food production environment in order to limit different food-borne pathogens such as bacteria, fungi and zoonotic viruses, the requirements to be effective against phages are especially challenging. First of all, in the case of the listed commercially available phage products (see Table 1), according to the manufacturer’s recommendations, about 10^8^ to 10^11^ (PFU/g, PFU/cm^2^, PFU/carcass, etc.; see Table S1) are usually applied. This would mean, that even a four to five log reduction due to disinfection would still leave 10^4^ PFU residues, which could persist in the food production environment [57,109,110,111]. Second, as mentioned before, the fact that most current commercial phage products use phages belonging to the order of *Caudovirales*, which are non-enveloped phages, casts some doubt regarding the efficacy of disinfectant measures usually designed against bacteria or enveloped viruses that are easier to inactivate.

Therefore, in the next two chapters of this review we will focus on possible limitations of disinfectant use in the food industry and afterwards on the current knowledge about the effectiveness of such disinfectants against different phages.

## 3. Legal Regulation of Disinfectants Currently Used in Food and Feed Processing Industry

As outlined in the previous chapter, there are an enormous variety of application fields for commercially available phage products (see Table 1, Tables S1 and S2).

Given the focus of this review on the food production environment, we will not discuss the possible disinfectant strategy for either pre-harvest phage applications as well as physical treatments (outlined in Figure 2) such as filtration, thermal inactivation or radiation. From our point of view these methods are not transferable towards efficient removal strategies against phages, especially in the dairy industry, and have been reviewed in depth elsewhere [77,80,81,151,152,153]. This leaves us with chemical disinfection measures and especially the use of sanitizers. The use of sanitizers, disinfectants and biocides is a common practice to control pathogens in the food industry [77,81,154]. Usually, cleaning in place (CIP) procedures are employed to remove organic materials and microbial contaminations from food contact surfaces [81]. Food contact sanitizers are applied after CIP to properly sanitize a surface [81]. Given the enormous amount of different regulations and a plethora of available sanitizer formulations, this review will only give a short overview on those substances, which are approved for food contact by the main issuing agencies: (i) The U.S. FDA, (ii) Health Canada (HC), (iii) the European Chemicals Agency (ECHA) (iv) the EFSA) (v) the United Nations Food and Agriculture Organization (FAO) and (vi) the World Health Organization (WHO) [155,156,157,158,159,160]. Due first to the detail and degree of information provided, second the different global areas covered and third the ease of accessibility.

For the classification of the various types of regulations, the FDA refers to the Code of Federal Regulations, Title 21 (“Food and Drugs”). In the EU, the ECHA maintains a database of biocidal active substances and categorizes 22 different product types of which only types four (“food and feed area”) and five (“drinking water”) are of relevance to this study. Thus, only substances with approval in these categories have respective entries in tables (see Table 2 and Table S4). Additional data on maximum residue limits (MRL) were obtained from the EU Pesticides Database for plants [161]. At the time of writing this manuscript, the EU Food contact material reference substances database (see Table S4) was under construction. MRL standards set by the WHO and FAO are summarized in Table S4 and were obtained from the Joint Committee on Food Additives and Contaminants (JECFA) database or the most recent Codex Alimentarius available online [162,163]. The level of permitted substance generally depends on the type of food and is regulated by the issuing agency. Here, agencies distinguish between maximum levels of use per application and maximum residue levels, while some of the most commonly used chemical disinfectants approved for food contact are alcohols, oxidative agents, some of which also affect pH and aldehydes.

### 3.1. Alcohols

Both ethanol and isopropanol have been approved by almost all agencies. However, the approval process for ethanol as an agent for food preservation and disinfection in the EU is in progress and awaiting an opinion by the Biocidal Products Committee (BPC) [157]. In addition, ethanol is under review for applications in human hygiene and animal feeds. With the US Environmental Protection Agency (EPA) [164], ethanol is GRAS (Generally Recognized As Safe) [165] approved and accepted as a multipurpose agent with antimicrobial properties but also as a solvent, processing aid, emulsifier and flavoring component. Furthermore, ethanol as an extractant is exempt from certification for select foods. Health Canada and the FAO/WHO list ethanol under carriers and extractants, and do not restrict maximum levels of use. Isopropanol is approved by the ECHA since 2016 for use in human hygiene products, disinfectants as well as in the food and feed area. The FDA lists a wide variety of permitted applications for isopropanol ranging from color additive to food additive and secondary additive permitted for direct addition to food. Unlike ethanol, however, isopropanol is not considered GRAS, for according to the WHO list there is no safety concern regarding isopropanol due to its relatively low toxicity and is approved as a flavoring agent as well as a carrier- and extraction solvent. Health Canada lists isopropanol as a carrier or extractant only with a maximum level limit of 50 ppm for natural extractive and spice extracts.

### 3.2. Aldehydes

Aldehydes such as glutaraldehyde or formaldehyde have been used extensively as disinfectants because of their broad spectrum of bactericidal, virucidal, fungicidal and sporicidal activity. Neither glutaraldehyde nor formaldehyde have entries in the HC or FAO/WHO databases. The FDA lists glutaraldehyde as an oxidizing/reducing agent and its concentration is limited to 250 ppm when used as a secondary direct food additive under certain conditions. In the European Union glutaraldehyde is a registered antimicrobial that may also be used on food and feed products [166]. In contrast to glutaraldehyde, formaldehyde is only permitted as a food additive in the United States with entries in the FDA database. However, the FDA strictly limits formaldehyde levels in direct food additives to a single role as part of defoaming agents.

### 3.3. Acids and Bases

The use of different acids or bases is widespread in various disinfectant formulations and it has often been reported that viruses are usually sensitive to low pH. Sulfuric acid is widely recognized as a food additive with listings at all four databases. Only at ECHA this substance is not yet approved but preregistered. With the FDA sulfuric acid is affirmed as GRAS and thus permitted in a variety of applications for food processing, primarily to adjust the pH but also as a flavor enhancer, processing aid, color additive and in sanitizing solutions. In this particular case, the maximum permitted concentration of sulfuric acid is limited depending on the type and quantity of other active components.

In a study by Sands et al. [167] it was demonstrated that fatty acids such as the oleic acid (C18:1) and palmitic acid (C16:0) have inhibitory properties against *Pseudomonas phaseolicola* phage Φ6, a surrogate for enveloped mammalian viruses. Fatty acids purified from extracts of *Rhodopseudomonas capsulata* could inhibit *Escherichia coli* phage T5, as reported by Takahashi et al. [168]. According to the authors the most potent compound was the linolelaidic acid (C18:2), which resulted in a 97% reduction of infectivity at a concentration of 50 µg/mL. All of the issuing agencies list at least one fatty acid as a permitted food additive. While HC only allows stearic acid in certain foods at GMP levels, the ECHA lists octanoic and decanoic acid as permitted food additives. Additionally, the EU pesticides database has entries for fatty acids covering the whole range from C_7_ up to C_20_ as approved. Both the FDA and the WHO/FAO have added a variety of fatty acids to their databases (see Table S4). Stearic, oleic and linoleic acid are GRAS approved by the FDA.

In Canada, sodium hydroxide is generally admitted as an additive to adjust the pH of foods at GMP levels, however, a limit of 70 ppm is given for the preparation of frozen crustaceans and molluscs in combination with sodium chloride or calcium oxide. The FDA lists sodium hydroxide as a GRAS certified multipurpose food additive with a wide spectrum of applications due to its low toxicity and its chemical properties as a potent reducing and/or pH control agent. For the treatment of food starch the FDA set a limit of one percent of the chemical used in solutions. FAO’s Codex Alimentarius lists sodium hydroxide as a pH regulator to be applied within GMP limits. Infant formulae are an exception with a maximum total sodium hydroxide concentration of 2 g/kg. Interestingly, sodium hydroxide has not been approved in the EU for use in the food/feed area or drinking water.

While trisodium phosphate is often used in formulations of cleaning agents and not per se as a disinfectant, it has also been reported as a potential virucidal substance [169,170,171]. There are no entries for trisodium phosphate in the ECHA database, but in other areas this phosphate salt is accepted with certain restrictions. For instance, HC limits the use of trisodium phosphate in/on cheeses in combination with a variety of other salts to 3.5% as anhydrous salt or 4.0% as total anhydrous salt, respectively. When added to select alcoholic beverages or unspecified foods the concentration limit for trisodium phosphate is GMP. The FDA lists trisodium phosphate as a GRAS certified food additive with a broad application spectrum ranging from anticaking/drying agent, emulsifier, humectant, sequestrant, pH control agent to nutrient supplement.

### 3.4. Chlorine and Chlorine Releasing Agents

Sodium hypochlorite, commonly known as “bleach”, is a very widespread chemical found in a plenitude of commercial household cleaners and disinfectants. It is accepted as a food additive in most areas except for WHO/FAO. In Canada, sodium hypochlorite may only be used in treatments of starch at GMP levels. Aside from its purpose listed as an adhesive, fumigant and antimicrobial, the US FDA allows adding sodium hypochlorite to food and feed products at GMP levels with certain restrictions, most of which are directed towards modifications of starch.

The strong oxidizing agents chlorine as well as chlorine dioxide are registered at the FDA as secondary direct food additives to be used only on flour and whole wheat flour within GMP limits. The same rules apply to these chemicals in Canada as set by HC as well as the FAO/WHO. For the US market, the FDA also lists chlorine dioxide as a food additive permitted in aqueous wash solutions with a given residual limit of 3 ppm. The allowed concentration of chlorine dioxide in sanitizer solutions ranges from 100 to 200 ppm.

### 3.5. Peroxides

Peracetic acid is generally accepted by all issuing agencies as a food additive. The FDA database lists four direct food additive entries for peracetic acid, two of which being secondary direct food additive applications. The chemical may be used as an oxidant in the production of food additives from hops, or as a bleaching agent in the treatment of starch. Small amounts of peracetic acid, up to a limit of 80 ppm in wash water, may be generated by the reaction of hydrogen peroxide with acetic acid during washing of vegetables or fruits. While HC lists peracetic acid only as a starch-modifying additive with GMP limits, its application is largely unrestricted in the European Union according to the ECHA [172]. Since peracetic acid is considered by the ECHA as chemically unstable, residual levels do not pose a safety concern to human consumption, and therefore an MRL is not given. Similarly, the WHO approved peroxyacetic acids as a whole as food additives with negligible safety concern due to the high rates of degradation of the chemicals into less toxic compounds.

Even though the peroctanoic acid is not registered as a food additive for the Canadian market according to the HC database, it has received approval by the FDA as well as the FAO/WHO. In Europe, the approval process for this antimicrobial is in progress. Similar to peracetic acid, peroctanoic acid is subject to regulations defined by the FDA for a broader group of peroxy acids. While the use of certain other peroxy acids as secondary direct food additives appears to be limited by the type of food, this is not the case for peroctanoic acid. As part of sanitizers, peroctanoic acid is considered an indirect food additive and its concentration is restricted to limits depending on the type of food contact surface.

Hydrogen peroxide is a commonly accepted, widely used oxidizing, bleaching and general-purpose antimicrobial agent. All issuing agencies have entries for this disinfectant due to its low degree of toxicity and the small environmental footprint. Its maximum permitted level is well documented for most food and feed related applications (see Table S4). HC lists several applications for hydrogen peroxide. When used in bleaches or in treatments of starch, HC’s limit is GMP, as a clarification aid in Brewers’ mash the total concentration in the mash, is limited to 135 ppm. A maximum of 100 ppm is set by HC when hydrogen peroxide is applied to whey protein for discoloration or pH adjustment. Hydrogen peroxide is considered GRAS by the FDA. Aside from its purpose as an antimicrobial and oxidizer in the food industry, the FDA also permits adding this disinfectant to dough strengtheners and fumigants. In bottled water, the maximum concentration is 23 mg/kg. In contrast to HC, the FDA particularly limits the amount of total active oxygen generated from hydrogen peroxide for the treatment of starch to 0.45%. Hydrogen peroxide may also be a secondary direct food additive and, for instance, be part of wash solutions. Here, the limit set by the FDA is GMP except when in combination with acetic acid where the maximum concentration of hydrogen peroxide is 59 ppm. Multiple restrictions apply for hydrogen peroxide according to the FDA database when this chemical is applied as antimicrobial or sanitizer. Maximum permitted concentrations depend on the type of food and food contact surface as well as on combinations with other chemicals.

### 3.6. Virucides Currently not Approved or Awaiting Approval

In addition to the range of disinfectants discussed in this work, there are of course other active substances currently applied in sanitizers, some of which are even currently under review for their suitability as food additives, (e.g., benzalkonium chloride by EFSA/ECHA) but could not be discussed in detail.

This includes active substances such as potassium peroxymonosulfate, ethoxylated nonylphenol, Triclosan, chlorhexidine diacetate, Quaternary Ammonium Compounds (QACs) such as benzalkonium chloride or ionic liquids, Chloramine-T and monochloramine, which have drawn the interest of research groups [41,169,171,173,174,175,176,177,178]. More information regarding the regulation of these substances is included in the supplemental material of this review (see Table S4).

## 4. Assessing the Virucidal Activity of Disinfectants against Phages

As outlined in the previous chapter, we are not only limited in terms of which disinfectants can be used in the food production environment, additionally there are serious concerns regarding the effectiveness of a given sanitizer against specific phages. While in the past the efficacy of disinfectants to inactivate viruses has been extrapolated solely from data based on testing against other microorganisms, particularly bacteria, it is nowadays clear that this was inadequate [58].

Today, there are many national recommendations for testing the virucidal activity such as the AFNOR (Association Française de Normalisation) in France, DVV (Deutsche Vereinigung zur Bekämpfung der Viruskrankheiten) in Germany and DEFRA (Department of Environment, Food and Rural Affairs) in the UK [190,191,192]. In North America, such recommendations come from Health Canada, the US Environmental Protection Agency (EPA) and the US FDA [155,156,164]. Generally, these recommendations include a two-step test evaluation in which the general virucidal activity of a particular virucide formulation is evaluated first with a suspension test protocol and subsequently with a test that simulates a field application. As the use of disinfectants in the food industry is strongly restricted, the question “Is my virucide working?” is becoming more and more important. However, as outlined before, the basis of this review is the need of an effective strategy in order to remove commercial phages applied for biocontrol measures from the food production facility or point-of-care (POC), while the legal recommendations have mainly focused on virucides for controlling and preventing the spread of viral diseases. Thus, the recommended test viruses have been chosen accordingly and none of the important phage orders used for biocontrol measures are included in those tests.

In general, viruses are considered to have a simple structure and are divided into families based size, capsid symmetry, type and form of nucleic acid and mode of replication [193,194]. Early work assessing the stability of infectious virus particles and their resistance to temperature changes was published almost a century ago by Tomaselli (1923), Nanavutty [195] and Krueger [196], followed by studies on phage resistance to various pH ranges by Sharp et al. [197] and Kerby et al. [198]. While the problem of antimicrobial resistance against disinfectants has received a lot of attention in the past decades, little is known about the possible resistance of different phages or viruses against virucides. Even for important human viruses, our understanding of the activity and the mechanisms of action of microbicidal chemicals remain quite fragmentary and information on the virucidal activity of antimicrobials is often extrapolated [58]. There is of course the realization that usually non-enveloped viruses are more stable against various stress conditions, (e.g., pH, thermal, pressure, desiccation, biocides or UV) compared to enveloped viruses and for the important human and animal viruses, these topics have been extensively reviewed elsewhere [58].

### 4.1. Phages as Models in Disinfectant Testing

As mentioned before, as a rule of thumb, it is known that enveloped viruses are more sensitive towards disinfectants than non-enveloped viruses. However, further generalizations are difficult, especially for non-enveloped viruses, as different sensitivities have already been reported for viruses belonging to the same group [199,200]. Unfortunately, in case of commercial phages applied in the food industry, little is known about the respective effectiveness of disinfectants. Up to date, there have been two main fields of inquiry where the efficacy against phage inactivation through antimicrobials was studied: (i) When they were used as surrogates for human viruses or animal viruses and (ii) to reduce the economic costs of fermentation failures due to phage infection.

The use of phages as surrogates for highly pathogenic or difficult to propagate viruses has a long tradition in the ecological, medical, veterinary and hygiene sciences, as phages are generally easy to cultivate, non-pathogenic to humans or animals but still display a high structural variability [58]. Probably the most frequently used model phages are PRD1 and MS2, which have been used extensively as model viruses in field and laboratory studies [201]. Phage MS2, a group I F-specific phage from the family of *Leviviridae*, is a non-enveloped single-stranded RNA virus with an icosahedral virion and a diameter of 26 nm. Due to its structural similarities, MS2 is often used as a model for poliovirus or human enteric viruses such as Hepatitis E virus and norovirus [58,202]. Phage PRD1 belongs to the family of *Tectiviridae* and is a non-enveloped double-stranded DNA virus with an icosahedral virion and with a diameter of 62 nm. PRD1 is considered a suitable surrogate for foodborne enteric viruses such as the human norovirus, adenovirus, and Hepatitis A virus [203,204]. Both phages have been extensively used for studying the transport and removal of pathogenic microorganisms in aquifers or water treatments [183,205,206,207,208] as well as studying the transmission and removal of viruses from surfaces or persons in different settings such as kitchens, health facilities or food surfaces [204,209,210].

Past studies regarding the efficacy and mode of action against viruses in general, sometimes also included phages while the focus always lay on important human or animal pathogenic viruses [58]. “Model” phages, which have been used for such disinfection studies include the coliphages MS2, T2, f2, λ2 or ΦX174, lactococcal phages P001 and P008 or the *Pseudomonas aeruginosa* phage PAO F116 [58,173,211,212,213,214,215,216]. According to these studies, for all the different disinfectants classes (Aldehydes, halogen-releasing agents, Biguanidines, QACs, Alcohols, Phenolics, Oxidizing agents, metallic salts, acids and others) there is almost no example where the effectivity of any such antimicrobial is not demonstrated against at least one particular phage [58,217,218]. Unfortunately, these results offer almost no information regarding the effectiveness of a particular disinfectant against commercial phages or its effectiveness in food settings. While it is clear that the efficacy of any particular antimicrobial depends on a number of factors, some inherent to their chemical nature, (e.g., concentration, pH, contact time, relative humidity), some inherent to the conditions on application, (e.g., type of surfaces, temperature, soiling). Unfortunately, most studies working with the classical model phages do not focus on evaluating the efficacy of virucides in food industry settings but more on health care settings [219]. Recent counterexamples are the studies of Chandler-Bostock et al. [220] as well as Morin et al. [173]. In the study of Chandler-Bostock et al. [220], the authors tested the efficacy of six commercial disinfectants against MS2 in the presence of high and low levels of organic matter to simulate the farm environment. The authors found that Iodophore-based disinfectants did not have a significant virucidal effect against MS2, while for peroxygen based disinfectants and glutaraldehyde-based disinfectants, the organic matter load made a significant difference in reducing efficacy, which has also been demonstrated for other disinfectants [217]. In that study, only a phenolic-based disinfectant was effective at all levels of organic matter concentrations. In the study of Morin et al. [173], the authors compared the virucidal efficacy of peracetic acid, potassium monopersulphate and sodium hypochlorite on phages P001 and MS2. The authors found that while sodium hypochlorite and potassium monopersulphate had similar phagicidal activities against P001 and MS2, the latter was resistant against peracetic acid for up to 55 times higher concentrations and the authors strongly recommend the need to validate the concentration of the disinfectant on highly representative models of the targeted viruses or phages.

Therefore, in the end the question remains, if the existing knowledge regarding the efficacy of virucides based on traditional model surrogate phages is readily transferrable towards the phages currently used for biocontrol measures. A field where this question has been of high relevance and has been intensively studied is the inactivation and removal of phages disturbing industrial fermentations.

### 4.2. Efficacy of Common Disinfectants against Naturally Occurring Phages

While there have been reports of phage infections in different industrial fermentation processes [13,67], most studies investigating the effectiveness of antiviral treatments to date have focused on strains of economically important lactic acid bacteria (LAB) phages. This is of course due to the fact, that LAB bacteria are being utilized in over two thirds of all commercial milk fermentations, and thus play a vital role in the production of fermented products such as cheeses, yogurt, buttermilk, and sour cream [221]. Most LAB phages belong to the family of *Siphoviridae*, which are part of the order of *Caudovirales*. This makes the knowledge obtained for these phages highly valuable concerning phages currently used for biocontrol measures. They are predominantly belonging to the family *Myoviridae* being also part of the order of *Caudovirales*. *Siphoviridae* are non-enveloped dsDNA phages, with a head-tail structure with the icosahedral head being about 60 nm in diameter and a non-contractile tail.

The use of sanitizers, disinfectants and biocides is common practice to control phages, particularly LAB phages in the food industry. In the dairy industry, cleaning in place (CIP) procedures are employed to remove organic materials and microbial contaminations from food contact surfaces. Food contact sanitizers are applied after CIP to properly sanitize a surface. As mentioned before, there has been a lot of work on the efficacy of those antimicrobials inactivating bacteria and viruses associated with human disease but the existing studies on inactivating phages paints a different picture.

Indeed, in a series of related studies from 1999–2012, the group of Reinheimer and Quiberoni investigated the efficacy of traditional disinfection treatments such as ethanol, isopropanol, peracetic acid and sodium hypochlorite against phages infecting important LAB bacteria such as *Streptococcus thermophilus*, *Lactococcus lactis*, *Lactobacillus delbrueckii*, *Lactobacillus helveticus*, *plantarum*, *casei* and *paracasei* [152,169,171,184,185,222,223]. Guglielmotti et al. [152] have previously published an extended summary of these studies. In short, the authors found that the different phages show varying susceptibility against classic disinfectants such as ethanol, isopropanol and sodium hypochlorite while the peracetic acid was found to be the best functional agent for phage inactivation.

Since then, there have been additional studies investigating the varying susceptibility of LAB phages against different virucides. For example, in the study of Campagna et al. [182], the authors performed an initial screening with the virulent lactococcal phage P008 investigating the efficacy of 23 commercial chemical products, including 21 food-grade sanitizers in the presence of 1% (*v*/*v*) whey. For each compound, two different concentrations in accordance to the manufactures recommendations and for two contact times (2 min and 15 min) were tested. The active ingredients of those food-grade chemicals included oxidizing agents, halogenated agents, alcohols, QACs, anionic acids, iodine-based acids, and one amphoteric compound. In their study, the authors found that chlorinated compounds, isopropanol, iodine-based compounds and the amphoteric compound were not effective for inactivating phage P008. Alcohols were also not very effective, although ethanol leads to a 4-log phage reduction after 15 min of contact time. All six peroxides, peracetic acid and acetic acid mixtures reached at least a 4-log unit reduction after 15 min at the low concentration or after 2 min at the high concentration. The two QACs and four anionic acids were the most effective in inactivating P008. In a subsequent test, the effectiveness of the five most effective sanitizers (two peroxide and peroxyacid mixtures, one QAC and two anionic acids) was evaluated against eight additional dairy phages belonging to the lactococcal 936, c2, Q54 and 1358 group as well as one *Lactobacillus* phage and one streptococcal phage in the presence of 1% (*v*/*v*) milk. While the authors found a higher resistance of the P1532 and CB13 lactococcal phages against the tested sanitizers (especially anionic acids), overall the peracetic acid and acetic acid mixtures as well as QACs ensured adequate inactivation of phages during sanitization of factories manufacturing fermented dairy products.

In a similar study, Murphy et al. [224] also described a large variation in the antiviral efficacy of sanitizers against eleven phages of the lactococcal 936-group. The authors found that, while peracetic acid (0.015%) and sodium hydroxide (0.2%) were effective against all tested phages, inactivation of lactococcal phages by sodium hypochlorite is phage-dependent and most of the eleven phages were not affected by exposure to 800 ppm sodium hypochlorite for 30 min. In the case of two commercial surface disinfectants (Virkon and Spor-Klenz), exposure of the eleven phages resulted in a complete loss of infective phage particles within a contact time of 10 min, while a generic disinfectant (alkyldimethylbenzylammonium chloride) failed to induce any phage inactivation. From their results, the authors concluded that the combination of several biocidal agents might be more effective in the elimination of dairy phages. In particular, especially the dominant 936-group phage, showed a significant robustness against especially sodium hypochlorite.

Probably the most comprehensive study of disinfectant effectiveness in regard to LAB phages was performed by Hayes et al. [12]. In this study, the authors investigated the susceptibility of 36,936-group phages to antimicrobial treatments using 14 antimicrobials and commercially available disinfectants. The authors investigated disinfectants commonly used in the food and beverage industries and included pure substances as well as industrial sanitizer solutions. Confirming the results of previous studies, the authors found that ethanol and isopropanol, but also oxidizers such as sodium percarbonate, sodium chlorite and sodium dichloroisocyanurate were ineffective against the phage collection. The most effective pure compound was found to be the QAC compound benzalkonium chloride, which eliminated all phages at 0.1% (*w*/*v*) after 30 min of incubation to undetectable limits. The oxidizer hydrogen peroxide was found to be less effective and required high concentrations (20% *w*/*v*) for complete elimination, while for the iodophor complex (Polyvinylpyrrolidone-iodine—PVP) a 4% (*w*/*v*) solution was required. Interestingly, at sublethal concentrations of each of these compounds, a wide variation between the different phages was found. Although all phages belong to the same group, the authors report that some of them were significantly more resistant to these compounds. Some were completely inactivated after 10 min, while other phages exhibited less than a single log reduction within 30 min. In the case of the industrial disinfectants, this study revealed high variability between chemical sanitizers and phages: While for example one QAC-based sanitizer (C8-C18 alkyl dimethyl chloride ammonium compound) was observed to be the most effective of the commercial sanitizers, even below the supplier’s recommended dilution. Another QAC based sanitizer (Ethanol 10%, chlor hexidinedigluconate 10%, Tetradecyl-trimethyl-ammonium-bromide <1%) was ineffective against the tested phages even at concentrations well above the supplier’s recommendations. The same was found to be true for two other industrial sanitizers based either on a Polymeric-biguanide-hydrochloride based or on a 30% nitric acid, 5% orthophosphoric acid based composition. In the case of two sanitizers with sodium hydroxide listed as their main active ingredient, a stable effectiveness well below the manufactures recommendations were found and the authors also conclude that such sanitizers are best suited as a reliable sanitizing agent against phages of this group. The authors concluded that large variations in resistance against disinfectants exist between phages and that phages resistant to biocidal activity tend to possess resistance to more than one compound.

### 4.3. Efficacy of Common Disinfectants against Commercial Phages

In case of commercial phages, to the best of our knowledge, so far there are no studies published, in which the topic of efficient removal of the respective phages after their application has been addressed. So far, most studies focus on stabilizing phages towards antimicrobial treatments in order for them to remain active. For example, Komora et al. [61] studied the stabilizing effect food matrices on Listeria lytic phage P100 towards high pressure processing while Meyer et al. [225] found that adsorption on paper can protect T-bacteriophages against pH stress. In the case of disinfectants, Agun et al. [41] investigated interactions between the antistaphylococcal phage phiIPLA-RODI and chemical disinfectants for synergistic or antagonistic effects for elimination of *Staphylococcus aureus* contamination. Although the elimination of the phage was not the focus of this study, the authors found differences between disinfectants (benzalkonium chloride, triclosan, chlorhexidine and hydrogen peroxide) regarding their effect on phage survival. Furthermore, the authors found that, with the exception of chlorhexidine, all disinfectants inactivated the phage particles in the suspension to undetectable levels after overnight incubation and at concentrations used in commercial products. In concentrations close to the MIC of *S. aureus*, only hydrogen peroxide still showed antiviral activity while the others were ineffective. In a similar study, Tomat et al. [186] studied the resistance of six lytic *Escherichia coli* phages (all belonging to the family of *Myoviridae*) against three commonly used disinfectants as well as five commercial sanitizers. The authors tested the viability of phages against different concentrations of the respective sanitizers for up to 24 h. As in the study by Agun et al. [41] the focus of this study was to find sanitizers, which are not effective against the respective phages, and the authors identified classical sanitizers such as ethanol and industrial antimicrobials based on quaternary ammonium chloride, hydrogen peroxide/peracetic acid/peroctanoic acid and p-toluenesulfonchloroamide to be compatible with the investigated phages. As a reverse conclusion, based on the results of this study, it could be concluded that peracetic acid, sodium hypochlorite or industrial sanitizers based on alkaline chloride foam or ethoxylated nonylphenol and phosphoric acid would be applicable to efficiently remove phages from the food production environment. Unfortunately, as this was not the focus of the study, the authors did not evaluate the efficiency of the disinfectants to inactivate the six phages under more realistic conditions such as different temperatures, presence of protein or fat and different surfaces.

Therefore, what is the conclusion we can draw from the existing literature in regard to choosing the right antimicrobial to remove commercial phages after successful application? Well, the truth is, we just do not know. Given the variety of applied commercial phages in combination with the knowledge about variable resistance of closely related LAB-phages, the current literature indicates that anything can be possible. It could definitely be true, that the existing CIP and sanitizing protocols applied in the respective food production facility are able to efficiently remove and inactivate the respective commercial phages. Given the current literature, this seems most probable for the peracetic acid based disinfectant formulations. However, it could also be true that the protocols are not effective against those phages. As a result, the phages would be allowed to persist in the respective food production facility or spread all along the food production chain and possibly lead to unwanted results such as increasing the risk of resistance development or interfere with the ability of diagnostic labs to monitor bacterial pathogens.

## 5. Future Perspectives

**Figure 3:**
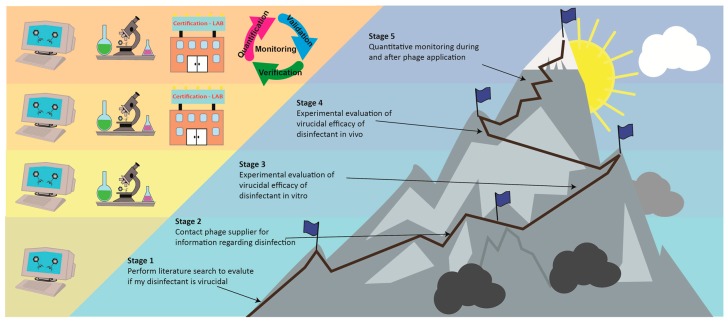
Inactivation and monitoring strategies: It´s a step climb to the top of phage mountain.

There is of course an easy way to stop this problem from ever occurring. While this review should highlight the fact that our knowledge about disinfectant activity against commercial phages is extremely scarce and has to be extended, also the manufacturers are inclined to act. Indeed, the advantage of the application of commercial products is that the companies know exactly what they are using and introducing into the food production facility. In contrast to LAB phages, huge evolutionary steps of phages developing resistance against disinfectants are thus not very likely. Therefore, it should be straightforward and easy for the companies to investigate and define applicable cleaning and disinfectant strategies for their products. Additionally, each user of commercial phage products should evaluate the steps they can do to ensure a safe and secure application in their facility and a possible strategy is outlined in Figure 3.

In a first step, the respective customer should research if their routine C&D measures have the potential to be virucidal either by a literature inquiry (stage 1) and/or by getting the respective information from their supplier (stage 2). The next step would be to evaluate the virucidal activity of their respective C&D measures against the respective phage product they intend to use in their facility. For this purpose, the respective ISO/CEN guidelines would provide a good strategy by testing the virucidal activity first in suspension tests (stage 3) before performing extensive and elaborate activity and surface testing (stage 4) [226]. The ultimate step would be to include a designated monitoring approach, already established for monitoring of pathogenic bacteria [227,228], in the routine monitoring system of the respective customer of phage products (stage 5). This would of course require the designated infrastructure to be present but ultimately would provide the security needed. Using this way, the customer would be able to validate and verify the effectiveness of their C&D measures by quantifying the remaining phage particles at the given point of application. Ideally, phage product manufacturers and distributors could also provide means and methods for efficient detection or quantification of their phage in order for food producers to perform an in-house efficiency testing of their CIP and effective disinfectant strategies.

In addition to adequate disinfectants against and efficient detection and monitoring systems for commercial phages [229], also a clear declaration system is needed, to increase the transparency of phage [230] treated food and feed. The increased transparency would also give an overview about the distributions of phage-using facilities, resulting in a better monitoring of passive phage spread and the development of phage-resistant mutants.

## 6. Conclusions

If one day pathogen-targeting phages are well established and broadly used in food production environments and on farms, hopefully it will be without making the same mistakes as with antibiotics. This includes not only the avoidance of overusing them, as it was exemplified with antibiotics, but also the development and monitoring of adequate removal of phages after their successful application.

As summarized in this review, it is still a long way to go to reach this goal. There is not only a need for ongoing research and development regarding successful inactivation and monitoring strategies, but it is also necessary to improve legislative provisions especially in view of labelling requirements. As time will tell, it is up to all people involved to demonstrate that we did learn from our previous mistakes with antibiotics and keep phages as a beneficial tool in the fight against bacterial pathogens.

## Figures and Tables

**Table 1:** List of all currently commercially available phage products sorted according to their field of application as a pre-harvest or post-harvest measure.

Pre-Harvest				Post-Harvest				
Target Organisms	Phage Product	Taxonomy	References	Target Organisms	Phage Product	Taxonomy	Application	References
*Escherichia coli* O157:H7	Ecolicide PX™ Finalyse®	*Caudovirales*	[112,113,114]	*Listeria monocytogenes* *	ListShield™ ListPhage™ PhageGuard Listex™	*Caudovirales: Myoviridae*	(Pet) Food Safety	[90,115,116,117,118]
*Salmonella*	PLSV-1™ BAFASAL®	/	[119,120]	*Escherichia coli* O157:H7*	EcoShield™ Ecolicide® PhageGuard E™ Secure Shield E1	*Caudovirales: Myoviridae, Podoviridae*	(Pet) Food Safety	[17,18,45,112,121]
*Clostridium perfringens*	INT-401™	*Caudovirales: Myoviridae, Siphoviridae*	[102,119]	*Salmonella* *	SalmoFresh™ SalmoLyse® PhageGuard S™ Salmonelex^TM^ SalmoPro®(2015) SalmoPro®(2018) Biotector® S1 Biotector® S4	*Caudovirales: Myoviridae, Podoviridae, Siphoviridae*	(Pet) Food Safety	[22,23,85,121,122,123]
*Vibrio parahemolyticus*	Lexia	*Caudovirales: Myoviridae*	[124,125,126]	*Shigella* spp.	ShigaShield™ (ShigaActive™)	*Caudovirales: Myoviridae, Siphoviridae*	Food Safety	[121,127,128]
*Xanthomonas campestris pv. vesicatoria* and *Pseudomonas syringe pv. tomato*	Agriphage™	*Caudovirales: Myoviridae*	[129,130,131,132]	*Staphylococcus, Streptococcus,* *Escherichia coli,* *Pseudomonas Aeruginosa, Proteus*	Pyo Bacteriophage	/	Pet Food Safety	[133,134]
*Clavibacter michiganensis* subsp. *michiganensis*	Agriphage™ CMM	*Caudovirales: Mycobacteriophage*	[135,136]	*Shigella,* *Salmonella,* *Escherichia coli,* *Proteus,* *Staphylococcus, Pseudomonas, Enterococcus*	Intesti Bacteriophage	/	Pet Food Safety	[133,137]
*Erwinia amylovora*	Agriphage™ FireBligth Erwiphage PLUS	*Caudovirales: Siphoviridae*	[138,139,140,141,142]	*Staphylococcus, Streptococcus,* *Escherichia coli*	SES Bacteriophage	/	Pet Food Safety	[133,143]
*Xanthomonas citri* subsp. citri	Agriphage™ CitrusCranker	*Caudovirales*	[112,138,144]	*Salmonellae, Shigella, Escherichia coli, Staphylococcus*	EnkoPhagum	/	Pet Food Safety	[133,145]
specific against soft rot *Enterobacteriacea*	Biolyse®-PB	*Caudovirales: Myoviridae*	[146,147]	*Staphylococcus Streptococcus*	Fersisi Bacteriophage	/	Pet Food Safety	[133,148]
*Pseudomonas* and *Aeromonas*	BAFADOR®	/	[9,149]	*Staphylococcal,* *Escherichia coli, Streptococcal, Pseudomonas aeruginosa, Proteus*	Mono-phage Preparations	/	Pet Food Safety	[133]

* Microorganisms which have to be monitored [150].

**Table 2:** Status of select disinfectants with issuing agencies.

Substance Class	Substance	CAS reg.	Canada (HC)	U.S. (FDA)	EU (ECHA)	FAO/WHO	References
Aldehydes	Glutaraldehyde	111-30-8	Not approved	Food additive	Approved	Not approved	[170,176,179]
Chlorine/Chlorine releasing agents	Chlorine	7782-50-5	Approved	Approved	Not approved *	Approved	[180,181]
	Chlorine dioxide	10049-04-4	Approved	Food additive	Under review *	Approved	[182,183]
	Sodium hypochlorite	7681-52-9	Approved	Food additive	Approved	Not approved	[169,182,184,185]
Peroxides	Hydrogen peroxide	7722-84-1	Food additive	Food additive, GRAS	Approved	Approved	[12,41]
	Peracetic acid	79-21-0	Food additive	Food additive	Approved	Approved	[169,184,185]
	Peroctanoic acid	33734-57-5	Not approved	Food additive	Approval in progress	Approved	[171,186]
Alcohols	Ethanol	64-17-5	Food additive	Food additive, GRAS	Approval in progress	Approved	[169,184,185]
	Isopropanol	67-63-0	Food additive	Food additive	Approved	Approved	[169,184,185]
Acids	Trisodium phosphate	7601-54-9	Food additive	Food additive, GRAS	Not approved	Approved	[169,170,171]
	Sulfuric acid	7664-93-9	Approved	Food additive, GRAS	Not approved; preregistered	Approved	[187]
	Sodium hypochlorite	7681-52-9	Food additive	Food additive	Approved	Not approved	[169,182,184,185]
	Different fatty acids	Various—see Supplement	Approved °	Approved °	Approved °	Approved °	[167,168,188]
Bases	Sodium bicarbonate	144-55-8	Food additive	Food additive, GRAS	Not approved; preregistered	Approved	[189]
	Sodium hydroxide	1310-73-2	Food additive	Food additive, GRAS	Not approved	Approved	[187]

* Direct application of the chemicals is currently not approved by the ECHA. Active chlorine and chlorine dioxide released from chlorine containing chemicals such as hydrochloric acid, hypochlorous acid, sodium chlorate, sodium chlorite and tetrachlorodecaoxide complex are being approved. The release of active chlorine from sodium hypochlorite is approved; Substances generally prohibited from use in human food in the U.S. are listed under the Electronic Code of Federal Regulations, Title 21 §189. ° Not all substances are approved; further information is listed in the Supplement.

## Supplementary Materials

Supplementary materials can be found at https://www.mdpi.com/1999-4915/11/5/468/s1.
