# Supplementary material for: Don’t Shut the Stable Door after the Phage Has Bolted—The Importance of Bacteriophage Inactivation in Food Environments
Source: Viruses. 2019 May 22;11(5):468. doi: 10.3390/v11050468 (PMC6563225; doi:10.3390/v11050468)
Supplement: Supplementary file 1 [file viruses-11-00468-s001.zip › Plain_Paper_Viruses_Supplement_20190521_Proof.docx]

Review

Don’t Shut the Stable Door after the Phage has Bolted—the Importance of Bacteriophage Inactivation in Food Environments

Julia Sommer ^1^, Christoph Trautner ^1^, Anna Kristina Witte ^1,3^, Susanne Fister ^4^, Dagmar Schoder ^2^, Peter Rossmanith ^1,2^ and Patrick-Julian Mester ^1,^*

^1^ Christian Doppler Laboratory for Monitoring of Microbial Contaminants, Department for Farm Animal and Public Health in Veterinary Medicine, University of Veterinary Medicine, Veterinaerplatz 1, 1210 Vienna, Austria; julia.sommer@vetmeduni.ac.at (J.S.); christoph.trautner@vetmeduni.ac.at (C.T.); anna.witte@vetmeduni.ac.at (A.K.W.); peter.rossmanith@vetmeduni.ac.at (P.R.)

^2^ Unit of Food Microbiology, Institute of Food Safety, Food Technology and Veterinary Public Health, Department for Farm Animal and Public Health in Veterinary Medicine, University of Veterinary Medicine, Veterinaerplatz 1, 1210 Vienna, Austria; dagmar.schoder@vetmeduni.ac.at

^3^ Current address: HTK Hygiene Technologie Kompetenzzentrum GmbH, Buger Str. 80, 96049 Bamberg, Germany

^4^ Former member of Christian Doppler Laboratory for Monitoring of Microbial Contaminants; susanne.fister1@gmail.com

***** Correspondence: patrick-julian.mester@vetmeduni.ac.at; Tel: +43-25077-3529; Fax: +43-25077-3590

Table S1. List of currently commercial available bacteriophage product used in the fields of agriculture, animal rearing and treatment and food and feed sector and their suggested application strategies.

| Company | Phage Product | Application | | | | | References |
| --- | --- | --- | --- | --- | --- | --- | --- |
|  |  | Usage on | Application type | Concentration | Time intervals | Field of application |  |
| Intralytix, Inc. (Baltimore, MD, USA) | ListShield™ | For direct food applications | Typically applied directly on food surfaces by spraying at a food product surface | Concentration of approximately 1 - 2 mL per 250 sq. cm | / | Post-Harvest -  Food Processing Facility | [1] |
|  | EcoShield™ | For direct food applications | Industrial sprayer for large direct food applications; handheld spryer for smaller bench-scale food applications | Diluted working solution - a concentration of 1 - 4 mL per pound of food product | / | Post-Harvest -  Food Processing Facility | [2] |
|  | SalmoFresh™ | For direct food applications  - Red meat and poultry | Industrial sprayer for large direct food applications; handheld spryer for smaller bench-scale food applications | Diluted working solution - a concentration of 1 - 4 mL per pound of food product | / | Post-Harvest -  Food Processing Facility sing Facility | [3] |
|  | ShigaShield™ (ShigaActive™) | Ready-to-eat meats, fish and shellfish, and fresh and processed fruits and vegetables, and dairy products | Direct application onto products | / | / | Post-Harvest -  Food Processing Facility | [4] |
|  | ListPhage™ | / | / | / | / | Post-Harvest –  Pet Food Safety | [5,6] |
|  | SalmoLyse® | / | / | / | / | Post-Harvest –  Pet Food Safety | [7,8] |
|  | Ecolicide® | / | / | / | / | Post-Harvest –  Pet Food Safety | [9-11] |
|  | Ecolicide PX™ | / | / | / | / | Pre-Harvest Interventions | [9-11] |
|  | PLSV-1™ | / | / | / | / | Pre-Harvest veterinary applications | [12] |
|  | INT-401™ | / | / | / | / | Pre-Harvest veterinary applications | [12,13] |
| Micreos Food Safety (Wageningen, Netherlands) | PhageGuard Listex™ | Surface ripened washed rinds and white mould cheeses;  ripening culture to protect the smearing robot against cross contamination during brushing and washing | Application of PhageGuard Listex is either by spraying (1ml per 100 cm2) or by immersion and is effective in solution | 0.5% (1x10^8^ ) - 1% (2x10^9^); in pasteurized milk or on the rind ,.. etc. | / | Post-Harvest -  Food Processing Facility | [14] |
|  | PhageGuard Listex™ | Whole fish, filets and in the environment | Spray onto product prior to packaging; Spray into package ; Spray onto slicer blade/dicer blades ; Dipping/immersion into a solution containing Listex | 2% (4x10^9^) | / | Post-Harvest -  Food Processing Facility | [15,16] |
|  | PhageGuard Listex™ | Meat products, RTE , frozen vegetables; products; meat slicer | Spray onto product prior to packaging; Spray into package ; Spray onto slicer blade/dicer blades ; Dipping/immersion into a solution containing Listex | 1% (2x10^9^) | / | Post-Harvest -  Food Processing Facility | [17,18] |
|  | PhageGuard Listex™ | \| Food-contact surfaces \| \| --- \| | Spray on the surface, add so much that the surface becomes wet | 1% dilution | Daily | Post-Harvest -  Food Processing Facility | [19] |
|  | PhageGuard Listex™ | \| Biofilms/Hot Spots treatments \| \| --- \| | \| Spray on the surface, add so much that the surface becomes wet \| \| --- \| | 5% dilution | \| Daily, until test negative \| \| --- \| | Post-Harvest -  Food Processing Facility | [19] |
|  | PhageGuard Listex™ | \| Food-processing environments \| \| --- \| | Spray on the surface, add so much that the surface becomes wet | 1% dilution | \| Weekly/Bi-weekly \| \| --- \| | Post-Harvest -  Food Processing Facility | [16,19] |
|  | PhageGuard S™ | Poultry | On line dipping / immersion /  Spray; Spray into a final package or Spray onto product prior to grinding | 1% dilution | / | Post-Harvest -  Food Processing Facility | [20] |
|  | PhageGuard E™ | Leafy green Vegetables | Using conventional spry or electrostatic spry or dipping | 3x10^7^ - 3x10^8^ PFU/cm^2^ | / | Post-Harvest -  Food Processing Facility | [21] |
|  | PhageGuard E™ | Beef carcass, parts and trim | Spray or Dip application | 3x10^7^ - 3x10^8^ PFU/cm^2^ | / | Post-Harvest -  Food Processing Facility | [22] |
|  | Salmonelex^TM^ | Pork and poultry products | / | levels up to I 0^8^ PFU/g offood | / |  | [23] |
| Passport Food Safety Solutions (West Des Moines, IA, USA) | Finalyse® | Cattle | Pre-harvest Hide Wash | 0.22% solution applied as an overhead spray system in the holding pens or lairage area spray system | Application timeline typically 1 to 1 ½ minutes | Pre-Harvest | [24,25] |
| OmniLytics  (Salt Lake City)  Phagelux  (Shanghai, China) Phagelux  (Montreal, Canada) | Agriphage™ | Tomato & Pepper | Greenhouse Seedling Treatment^1^ | 1 pint of AgriPhage per 50-100 gallons of water per 9600 square feet of greenhouse space | Apply daily treatments to foliage by spraying or fogging | Agriculture -  Pre-Harvest | [26] |
|  |  | Tomato & Pepper | Field Treatment – Ground Application^1^ | 1 to 2 pints of AgriPhage per 50-100 gallons of water per acre | Repeat application 1-3 times per week | Agriculture -  Pre-Harvest | [26] |
|  | Agriphage™ &  Agriphage™ CMM | Tomato & Pepper | Field Treatment – Aerial Application | 1-2 pints per acre in a minimum of 5 gallons of water per acre | / | Agriculture -  Pre-Harvest | [26,27] |
|  | Agriphage™ CMM | Tomato | Greenhouse Seedling Treatment^1^ | 1 pint of AgriPhage-CMM per 12-25 gallons of water per 9600 square feet of greenhouse space | Apply daily treatments to foliage by spraying or fogging | Agriculture -  Pre-Harvest | [27] |
|  |  | Tomato | Greenhouse Treatment – For Tomatoes Grown Hydroponically^1^ | to 1 quart of AgriPhage-CMM per 12-25 gallons of water per 9600 square feet of greenhouse space | Apply to foliage by spraying or fogging.  Repeat application 1-3 times per week | Agriculture -  Pre-Harvest | [27] |
|  |  | Tomato | Field Treatment – Ground Application^1^ | 1 to 2 pints of AgriPhage-CMM per 50-100 gallons of water per acre | Repeat application 1-3 times per week | Agriculture -  Pre-Harvest | [27] |
|  | Agriphage™ FireBligth | Apples & Pears | Field Treatment – Ground Application^1^ | 1 to 2 quarts of  AGRIPHAGEFIRE BLIGHT  per 50-100  gallons of  water per acre. | Repeat application weekly or as needed. | Agriculture -  Pre-Harvest | [28] |
|  | Agriphage™ CitrusCranker | Citrus e.g., orange, grapefruit, pummelo, lemon, lime, tangerine, tangelo, or kumquat | Field Treatment – Ground Application^1^ | 1 to 2 quarts of  AGRIPHAGECITRUS  CANKER per  50-100 gallons  of water per acre | Repeat application weekly or as needed | Agriculture -  Pre-Harvest | [29] |
|  | Lexia | *Penaeus vannamei* shrimp | „In water“ -Treatment | / | / | Aqua-culture | [30] |
|  | SalmoPro® (2015) | Food | / | Application rate at a maximum of 1 x 10^8^ PFU/g of food | / | Post-Harvest -  Food Processing Facility | [23] |
|  | SalmoPro® (2018) | Poultry, Red meat, Fruit, Vegetables, Eggs, Fish and shellfish | / | Application of SalmoPro^®^ at a maximum rate of 1x10^8^ PFU/g of food | / | Post-Harvest -  Food Processing Facility | [4] |
|  | Armament™ | Poultry and cattle | Application as a spray mist or wash on the feathers of live poultry prior to slaughter to decrease pathogen transfer to meat | / | / | Pre-Harvest | [23,31] |
| [Enviroinvest Környezetvédelmi és Biotechnológiai Zrt.](https://www.facebook.com/enviroinvest/) | Erwiphage PLUS | Apple | Application as a spray | / | During flowering periode | Agriculture -  Pre-Harvest | [32] |
| APS Biocontrol Ltd | Biolyse®-PB | Potatoes | Application via spray-bar equipment | / | / | Post-Harvest -  Food Processing Facility | [33] |
| CheilJedang Corporation | Biotector® S1 | Animal feed | Replace antibiotics in animal feed | / | / | Pre-Harvest  - Animal Breeding | [23] |
|  | Biotector® S4 | Animal feed | Replace antibiotics in animal feed | / | / | Pre-Harvest  - Animal Breeding | [23] |
| FINK TEC GmbH (Hamm, Germany) | Secure Shield E1 | Meat processing facilities; Beef carcasses | Spray systems in meat processing facilities | Approximately 1.5x10^11^ phage particles per carcass | / | Post-Harvest -  Food Processing Facility | [34] |
| Proteon Pharmaceuticals | BAFADOR® | Commercial aquaculture, fishes | „In water“ -Treatment | / | / | Pre-Harvest  - Animal Breeding | [35] |
|  | BAFASAL® | Poultry | Feed additive that eliminates, or prevents, salmonella infection in the digestive tract in poultry | Applied by being added to the drinking water | / | Pre-Harvest  - Animal Breeding | [36] |
| Brimrose Technology Corporation | Pyo Bacteriophage | / | Used in the treatment and prophylaxis of purulent inflammatory and enteric infectious diseases | / | / | Post-Harvest –  Pet Food Safety | [37,38] |
|  | Intesti Bacteriophage | / | Can be used in therapeutic and prophylactic purposes in pyo-inflammatory and enteric infections | / | / | Post-Harvest –  Pet Food Safety | [37,39] |
|  | SES Bacteriophage | / | Can be used in therapeutic and prophylactic purposes in pyo-inflammatory and enteric infections | / | / | Post-Harvest –  Pet Food Safety | [37,40] |
|  | EnkoPhagum | / | Can be used in therapeutic and prophylactic purposes in pyo-inflammatory and enteric infections | / | / | Post-Harvest –  Pet Food Safety | [37,41] |
|  | Fersisi Bacteriophage | / | Can be used in therapeutic and prophylactic purposes in pyo-inflammatory and enteric infections | / | / | Post-Harvest –  Pet Food Safety | [37,42] |
|  | Mono-phage Preparations | / | / | / | / | Post-Harvest –  Pet Food Safety | [37] |
|  | Staphylococcal Bacteriophage | / | Used in the treatment and prophylaxis of infections | / | / | / | [43] |
| ^1^ AgriPhage may be applied up to and including the day of harvest. | | | | | | | |

Table S2. List of currently commercial available bacteriophage product used in the fields of agriculture, animal rearing and treatment and food and feed sector.

| Company | Phage Product | Target Organisms | Used Phages | Taxonomy | Application | Reference |
| --- | --- | --- | --- | --- | --- | --- |
| Intralytix, Inc. (Baltimore, MD, USA) | ListShield™ | *Listeria monocytogenes* | Six-phages cocktail | Caudovirales | Food Safety | [44] |
|  | EcoShield™ | *Escherichia coli* O157:H7 | Three-phages cocktail | Caudovirales | Food Safety | [45] |
|  | SalmoFresh™ | pathogenic Salmonella-Serotypes; *Salmonella spp.* | Six-phages cocktail; | Caudovirales | Food Safety | [45,46] |
|  | ShigaShield™ (ShigaActive™) | *Shigella spp.* | Five-phage cocktail; SHFML-11, SHFML-26, SHSML-45, SHBML-50-1, SHSML-52-1 | Caudovirales: Myoviridae | Food Safety | [45,47,48] |
|  | ListPhage™ | *Listeria monocytogenes* | / | Caudovirales | Pet Food Safety |  |
|  | SalmoLyse® | *Salmonella* | / | Caudovirales | Pet Food Safety |  |
|  | Ecolicide® | *Escherichia coli* O157:H7 | / | Caudovirales | Pet Food Safety |  |
|  | Ecolicide PX™ | *Escherichia coli* O157:H7 | / | Caudovirales | Pre-Harvest Interventions |  |
|  | PLSV-1™ | *Salmonella* | / | / | Phage preparations for veterinary applications | [12] |
|  | INT-401™ | *Clostridium perfringens* | Five bacteriophages (CPAS-7, CPAS-12, CPAS-15, CPAS-16  and CPLV-42) | Caudovirales: Myoviridae, Siphoviridae | Phage preparations for veterinary applications | [12,13] |
| Micreos Food Safety (Wageningen, Netherlands) | PhageGuard Listex™ | *Listeria monocytogenes* | Single phage; P100 | Caudovirales: Myoviridae | Food Safety | [14,15,17] |
|  | PhageGuard S™ | *Salmonella spp.* | / | Caudovirales | Food Safety | [20,46] |
|  | PhageGuard E™ | *Escherichia coli* O157:H7 | / | Caudovirales | Food Safety | [21,22,49] |
|  | SalmonelexTM | *Salmonella* serovars | S16 and FO1a | Caudovirales: Myoviridae | Food Safety | [23,50] |
| Passport Food Safety Solutions (West Des Moines, IA, USA) | Finalyse® | *Escherichia coli* O157:H7 | / | Caudovirales | Pre-Harvest hide wash | [24,25,49] |
|  | Lexia | *Vibrio parahemolyticus* | Three-phages cocktail: VP7, VP46, VP48 | Caudovirales: Myoviridae | Aquaculture | [30,51,52] |
| OmniLytics  (Salt Lake City)  Phagelux  (Shanghai, China)    Phagelux  (Montreal, Canada) | Agriphage™ | *Xanthomonas campestris* pv. *vesicatoria* and *Pseudomonas syringe* pv. *tomato* | Phage phil7; 6-8 phages | Caudovirales: Myoviridae | Agriculture | [26,53-55] |
|  | Agriphage™ CMM | *Clavibacter michiganensis* subsp. *michiganensis* | / | Caudovirales: Mycobacteriophage | Agriculture | [27,56] |
|  | Agriphage™ FireBligth | *Erwinia amylovora* | / | Caudovirales | Agriculture | [28,57,58] |
|  | Agriphage™ CitrusCranker | *Xanthomonas citri* subsp. *citri* | / | Caudovirales | Agriculture | [29,49,59] |
|  | SalmoPro® (2015) | *Salmonella enterica* | Two-phage cocktail: BP-63 and BP-12 | Caudovirales: Myoviridae, Siphoviridae,  Podoviridae | Food Safety | [23] |
|  | SalmoPro® (2018) | *Salmonella enterica* | Two-phage cocktail: BP-63 and LVR16-A | Caudovirales: Myoviridae, Siphoviridae | Food Safety | [4] |
|  | Armament™ | *Salmonella spp.* | / | / | Food Safety | [23,31] |
| [Enviroinvest Környezetvédelmi és Biotechnológiai Zrt.](https://www.facebook.com/enviroinvest/) | Erwiphage PLUS | *Erwinia amylovora* | PhiEaH2, PhiEaH1 | Caudovirales: Siphoviridae | Agriculture | [32,57,58,60] |
| APS Biocontrol Ltd | Biolyse®-PB | Specific against soft rot Entero-bacteriacea | / | Caudovirales: Myoviridae | Agriculture; Food Safety | [33,61] |
| CheilJedang Corporation | Biotector® S1 | *Salmonella Galliinarum* (SG) and *S. Pullorum* (SP) | / | / | Pet Food Safety | [23] |
|  | Biotector® S4 | *Salmonella Galliinarum* (SG) and *S. Pullorum* (SP) | / | / | Pet Food Safety | [23] |
| FINK TEC GmbH (Hamm, Germany) | Secure Shield E1 | *E. coli* & *E. coli* O157:H7 | (AB27), (TB49), (TB120), (KRA2), (TB69), (BO1), (EW2), (TB6A), (GWF), (HAM53), (MP57), (TB11) | Caudovirales: Myoviridae, Podoviridae | Food Safety | [34] |
| Proteon Pharmaceuticals | BAFADOR® | *Pseudomonas* and *Aeromonas* | / | / | Pre-Harvest Interventions Aquaculture | [35,62] |
|  | BAFASAL® | *Salmonella* | / | / | Pre-Harvest Interventions | [36] |
| Brimrose Technology Corporation / Eliava Authorized Pharmacy | Pyo Bacteriophage | Staphylococcus (*S. aureus*), Streptococcus (*S. pyogenes*, *S. sanguis*, *S. salivarius*, *S. agalactiae*), *E.coli* (Different types), *Pseudomonas aeruginosa*, *Proteus mirabilis* and *P. vulgaris* | 5 components/phages | / | Pet Food Safety | [37,38] |
|  | Intesti Bacteriophage | *Shigella flexneri* (Serotypes 1,2,3,4; *Shigella sonnei*; *Shigella newcastle*; *Salmonella paratyphi A* and *Salmonella paratyphi B*; *Salmonella typhimurium*; *Salmonella enteritidis*; *Salmonella choleraesuis*; *Salmonella oranienburg*; *Escherichia coli*; *Proteus vulgaris*, *P. mirabilis*; *Staphylococcus aureus*; *Pseudomonas aeruginosa*; *Enterococcus faecalis* | 17 components/phages | / | Pet Food Safety | [37,39] |
|  | SES Bacteriophage | Staphylococcus (*S. aureus*, S*. epidermidis*), Streptococcus (*S. pyogenes*, *S. sanguis*, S*. salivarius*, *S. agalactiae*); different types of *E.coli* | 3 components/phages | / | Pet Food Safety | [37,40] |
|  | EnkoPhagum | S*almonellae* [Paratyphus A, Paratyphus B, Typhimurium, Enteritidus, Choleraesuis, Oranienburg, Dublin, Anatum], *Shigellas* [Flexner, Zonne], Enterepathogenic serotypes of *Escherichia coli* [10 types], *Staphylococcus* [3 types]) | 23 components/phages | / | Pet Food Safety | [37,41] |
|  | Fersisi Bacteriophage | Staphylococcus (*S. aureus*, *S. epidermidis*) Streptococcus (*S. pyogenes*, *S. sanguis*, *S. salivarius*, *S. agalactiae*) | 7 components/phages | / | Pet Food Safety | [37,42] |
|  | Mono-phage Preparations | Staphylococcal, *E. coli*, Streptococcal, *Pseudomonas aeruginosa*, Proteus | 5 components/phages | / | Pet Food Safety | [37] |
| Eliava Authorized Pharmacy | Staphylococcal Bacteriophage | *Staphylococcus aureus* | / | / | / | [43] |

Table S3. List of patents of bacteriophages submitted by commercial phage product manufacturers for usage in food environments.

| Company | Patent | Target Organisms | Used Phages | Taxonomy | Application | Reference |
| --- | --- | --- | --- | --- | --- | --- |
| Intralytix, Inc. (Baltimore, MD, USA) | Patent No.: US 8,685,697 B1 | *Listeria monocytogenes* | Patent 2014: LMSP-25, LMTA-34, LMTA-57, LMTA-94, or LMTA-148; | Caudovirales | Food Safety | [5] |
|  | Patent No.: US 7,507,571 B2 | *Listeria monocytogenes* | Patent 2009: List 1, List 2, List3, List 4, List 36 and List38 | Caudovirales | Food Safety | [6] |
|  | Patent No.: US 7.625,556 B2 | *Escherichia coli* O157:H7 | Patent 2009: ECTA-47, ECML-83, ECML-119, and ECML-122; | Caudovirales | Food Safety | [9] |
|  | Patent No.: US 7,635,584 B2 | *Escherichia coli* O157:H7 | Patent 2009_2: ECML-4 | Caudovirales | Food Safety | [10] |
|  | Patent No.: US 7.625,741 B2 | *Escherichia coli* O157:H7 | Patent 2009_1: ECML-117 and ECML-134 | Caudovirales | Food Safety | [11] |
|  | Patent No.: US 7,674.467 B2 | pathogenic Salmonella-Serotypes; *Salmonella spp.* | SPT-1, SBA-178, SBA-1781, SIT 128, SSE-121 and SDT-15 | Caudovirales | Food Safety | [7] |
|  | Patent No.: US 8,685,696 B2 | pathogenic Salmonella-Serotypes; *Salmonella spp.* | STML-198, SNN-387, SEML-239-1, STML-13-1,SKML-39. SEML-24, and STA-202 | Caudovirales | Food Safety | [8] |
|  | Patent No.: US 2016/0215273 A1 | *Shigella spp.* | SHFML-26, SHFML-11, SHSML-45, SHSML-52-1, SHBML-50-1, SHBML-50-2, SHSML-52-2, SHSML-36, and SHFML-21 | Caudovirales: Myoviridae | Food Safety | [63] |

Table S4. Status of select biocides with issuing agencies and permitted maximum levels.

| Substance class | Substance | Hazards | CAS reg. # | Canada (HC) | U.S. (FDA) | EU (ECHA) | FAO/WHO | References |
| --- | --- | --- | --- | --- | --- | --- | --- | --- |
| Aldehydes | Glutaraldehyde | C,A, I, H, E | 111-30-8 | Not approved | Food additive/ Permitted maximum levels of oxidizing/reducing agent: 250 ppm | Approved,  Regulation (EU) 2015/1759 | Not approved | [64-66] |
|  | Formaldehyde | F, C, I, H | 50-00-0 | Not approved | Food additive | Not approved | Not approved | [67,68] |
|  | Ethylene oxide | F, G, A, I, H | 75-21-8 | Not approved, under regulation by PCPA | Food additive | Not approved | Not approved | [69] |
| Chlorine and chlorine releasing agents | Chlorine | O, G, A, I, E | 7782-50-5 | Approved for flour and whole wheat flour only; Permitted maximum levels for chlorine gas: | Flour and whole wheat flour only; Permitted maximum levels for chlorine gas: GMP | Not approved * | Approved for flour only; FAO: 2.5 g/kg (treatment) for flour only  WHO: 2.5 g/kg (treatment) for flour only | [70,71] |
|  | Chlorine dioxide | O, G, C, A, I, E | 10049-04-4 | Approved for flour and whole wheat flour only; Permitted maximum levels: GMP | Food additive; Permitted maximum levels: GMP; antimicrobial wash solutions: 3 ppm  sanitizers: 100 ppm (minimum), 200 ppm (maximum) | Under review * | Approved for flour only; Permitted maximum levels: WHO: 30 mg/kg (treatment), 75 mg/kg (conditional) for flour only | [72,73] |
|  | Sodium hypochlorite | C, I, E | 7681-52-9 | Approved, permitted on starch only; Permitted maximum levels: GMP | Food additive; Permitted maximum levels: GMP except starch  residual total chlorine: 0.055 pds per pd dry starch, 0.0082 pds per pd dry starch when used as bleaching agent | Approved,  Regulation (EU) 2017/1273; Permitted maximum levels: 10 µg per kg according to  Regulation (EC) No 396/2005 | Not approved | [72,74-76] |
|  | Monochloramine | C, I, H | 10599-90-3 | Not approved | Not approved | Approval for drinking water in progress | Not approved | [77] |
|  | Chloramine-T | C, I, H | 127-65-1 | Not approved | Not approved | Approval in progress | Not approved | [67] |
| Iodophores | Polyvinlylpyrrolidine-iodine | C, I, E | 25655-41-8 | Not approved | Not approved | Approved | Not approved | [78] |
| Peroxides | Hydrogen peroxide | O, C, I | 7722-84-1 | Food additive; Permitted maximum levels: bleaching agents and starch: GMP  liquid whey: 100 ppm  brewers’ mash: 135 ppm residual | Food additive, GRAS; Permitted maximum levels: bottled water: 23 mg/kg  food starch: 0.45 % active residual oxygen from hydrogen peroxide  wash water: 59 ppm  hydrogen peroxide solution: 35 %  21CFR184.1366 | Approved,  Regulation (EU) 2015/1730; Permitted maximum levels: according to Regulation (EU) 2017/1777 (Annex IV) for hydrogen peroxide | Approved; Permitted maximum levels: WHO: unspecified amounts of residue from wash solutions are permitted | [78,79] |
|  | Potassium peroxymonosulfate | O,C, I, H | 37222-66-5 | Not approved | Not approved | Not approved | Not approved | [80] |
|  | Peracetic acid | F, C, A, I, E | 79-21-0 | Food additive; Permitted maximum levels: for starch: GMP | Food additive; Permitted maximum levels: residual on fruits: 80 ppm  antimicrobial agent: 220 ppm | Approved,  Regulation (EU) 2016/672 | Approved (peroxyacetic acids); Permitted maximum levels: no safety concern due to degradation into compounds of low toxicity | [74-76] |
|  | Peroctanoic acid | F, C, E | 33734-57-5 | Not approved | Food additive; Permitted maximum levels: poultry carcasses, poultry parts and organs: GMP  in sanitizers: restrictions apply depending on the application and type of food contact surface | Approval in progress | Approved | [81,82] |
| Phenols | Nonylphenols | C, I, E | 9016-45-9 | not approved | Not approved | Not approved | Not approved | [82] |
|  | 5-Chloro-2-(2,4-dichlorophenoxy)phenol (Triclosan) | I, E | 3380-34-5 | Not approved | Not approved | Not approved | Not approved | [79] |
| Quarternary ammonium compounds | Benzalkonium chloride | C, I, E | 8001-54-5 | Not approved | Not approved | Under review,  Regulation (EU) 1119/2014 | Not approved | [75,78,79] |
| Alcohols | Ethanol | F, I, H | 64-17-5 | Food additive; Permitted maximum levels: carrier/extractant: GMP | Food additive, GRAS | Approval in progress | Approved; Permitted maximum levels: GMP | [74-76] |
|  | Isopropanol | F, I | 67-63-0 | Food additive; Permitted maximum levels: carrier/extractant: 50 ppm  residual in fish protein: 0.15% | Food additive | Approved, Regulation (EU) 2015/407 | Approved; Permitted maximum levels: no safety concern due to low toxicity | [74-76] |
| Acids | Trisodium phosphate | C, I | 7601-54-9 | Food additive; Permitted maximum levels: alcoholic beverages: GMP  cheeses: 3.5% as anhydrous salt or 4% total anhydrous salt each when in combination with other salts | Food additive, GRAS; Permitted maximum levels: steam: GMP | Not approved | Approved; Permitted maximum levels: FAO: limits for total residual phosphorous: 3.52 g/kg (humectants), 1.32 g/kg (emulsifiers) | [64,75,82] |
|  | Sulfuric acid | C | 7664-93-9 | Approved; Permitted maximum levels: starch: GMP | Food additive, GRAS; Permitted maximum levels: caramel, starch: GMP  in sanitizers: varies with type and concentration of other components | Not approved but preregistered; Permitted maximum levels: not required according to Regulation (EU) No 1146/2014 | Approved; | [83] |
|  | Sodium hypochlorite | C, I, E | 7681-52-9 | Food additive, permitted on starch only; Permitted maximum levels:starch: GMP | Food additive | Approved,  Regulation (EU) 2017/1273 | Not approved | [72,74-76] |
|  | Fatty acids | varies | 124-07-2 (C8:0),  334-48-5 (C10:0),  143-07-7 (C12:0),  544-63-8 (C14:0),  57-10-3 (C16:0),  57-11-4 (C18:0),  112-80-1 (C18:1),  60-33-3 (C18:2) | Not approved except C18:0; Permitted maximum levels: GMP | Approved: C8:0, C10:0, C12:0, C14:0, C16:0,  C18:0 (GRAS), C18:1 (GRAS), C18:2 (GRAS) | Approved: C7-C20; Permitted maximum levels: GMP | Approved: C8:0, C10:0, C12:0, C14:0, C16:0, C18:0, C18:1, C18:2; Permitted maximum levels: GMP | [84-86] |
| Bases | Sodium bicarbonate | I | 144-55-8 | Food additive; Permitted maximum levels: Unstandardized confectionery, starch and salt: GMP | Food additive, GRAS | Not approved but preregistered | Approved; Permitted maximum levels: FAO: generally GMP except for infants: 2 g/kg | [87] |
|  | Sodium hydroxide | C, I | 1310-73-2 | Food additive; Permitted maximum levels: when used together with sodium chloride and calcium oxide: 70 ppm | Food additive, GRAS; Permitted maximum levels: caramel: GMP  food starch: 1 % | Not approved | Approved; Permitted maximum levels: FAO: generally GMP except for infants: 2 g/kg | [83] |
| Biguanides | Chlorhexidine diacetate | A, I, E | 56-95-1 | Not approved | Not approved | Not approved | Not approved | [66,79] |
| Ionic liquids | Alkyl methylimidazole chloride | Varies | n.a. | Not approved | Not approved | Not approved | Not approved | [88-90] |
| * Direct application of the chemicals is currently not approved by the ECHA. Active chlorine and chlorine dioxide released from chlorine containing chemicals such as hydrochloric acid, hypochlorous acid, sodium chlorate, sodium chlorite and tetrachlorodecaoxide complex are being approved. The release of active chlorine from sodium hypochlorite is approved; Substances generally prohibited from use in human food in the U.S. are listed under the Electronic Code of Federal Regulations, Title 21 §189. | | | | | | | | |
| C ... Corrosive; A ... Acute toxicity; I ... Irritant; H ... Health Hazard; E ... Environment toxicity; F ... Flammable; G ... compressed Gas | | | | | | | | |

References

1. Intralytix. Listshieldtm. <http://www.intralytix.com/files/prod/01LP/01LP-Desc.pdf> (accessed on 25 January 2019).
2. Intralytix. Ecoshieldtm. Available online: <http://www.intralytix.com/files/prod/07EP/07EP-Desc.pdf> (accessed on 25 January 2019).
3. Intralytix. Salmofreshtm. Available online: <http://www.intralytix.com/files/prod/02SP/02SP-Desc.pdf> (accessed on 25 January 2019).
4. Phagelux-Inc. Gras notification: Salmopro®. Available online: <http://www.fda.gov/downloads/Food/IngredientsPackagingLabeling/GRAS/NoticeInventory/UCM624100.pdf> (accessed on 25 January 2019).
5. Pasternack, G.R.; Sulakelidze, A. *Listeria monocytogenes* bacteriophages and uses thereof. *Intralytix, Inc., Baltimore, MD (US)* **2014**.
6. Pasternack, G.R.; Sulakelidze, A. *Listeria monocytogenes* bacteriophages and uses thereof. *Intralytix, Inc., Baltimore, MD (US)* **2009**.
7. Sulakelidze, A.; Sozhamamnnan, S.; Pasternack, G.R. *Salmonella* bacteriophage and uses thereof. *Intralytix, Inc., Baltimore, MD (US)* **2010**.
8. Pasternack, G.; Sulakelidze, A. *Salmonella* bacteriophage and uses thereof. *Intralytix, Inc., Baltimore, MD (US)* **2014**.
9. Sulakelidze, A.; Pasternack, G.R. *E. Coli* bacteriophage and uses thereof. *Intralytix, Inc., Baltimore, MD (US)* **2009**.
10. Sulakelidze, A.; Pasternack, G.R. *E. Coli* O157:H7 bacteriophage and uses thereof. *Intralytix, Inc., Baltimore, MD (US)* **2009**.
11. Pasternack, G.R.; Sulakelidze, A. *E. Coli* O157:H7 bacteriophage and uses thereof. *Intralytix, Inc., Baltimore, MD (US)* **2009**.
12. Intralytix. Veterinary applications. Available online: <http://www.intralytix.com/index.php?page=vet> (accessed on 13 February 2019).
13. Miller, R.W.; Skinner, E.J.; Sulakvelidze, A.; Mathis, G.F.; Hofacre, C.L. Bacteriophage therapy for control of necrotic enteritis of broiler chickens experimentally infected with C*lostridium perfringens*. *Avian Dis.* **2010**, *54*, 33-40.
14. Micreos-Food-Safety-BV. Phageguard listex application data sheet cheese. Available online: <http://www.phageguard.com/wp-content/uploads/2017/03/PhageGuard-Listex-Application-Data-Sheet-Cheese-Final.pdf> (accessed on 25 January 2019).
15. Micreos-Food-Safety-BV. Phageguard listex application data sheet: Salmon. Available online: <http://www.phageguard.com/wp-content/uploads/2017/03/PhageGuard-Listex-Aplication-Data-Sheet-Salmon-March-17.pdf> (accessed on 25 January 2019).
16. Micreos-Food-Safety-BV. How phages can help reduce listeria risks on salmon. Available online: <http://www.phageguard.com/wp-content/uploads/2018/11/PhageGuard-whitepaper-Salmon.pdf> (accessed on 25 January 2019).
17. Micreos-Food-Safety-BV. Phageguard listex application data sheet meat. Available online: <http://www.phageguard.com/wp-content/uploads/2017/03/PhageGuard-Listex-Aplication-Data-Sheet-RTE-Meat-FINAL.pdf> (accessed on 25 January 2019).
18. Micreos-Food-Safety-BV. Phageguard-listex reduces *Listeria* on frozen vegetables. Available online: <http://www.phageguard.com/wp-content/uploads/2017/11/PhageGuard-ADS-Frozen-Vegetables.pdf> (accessed on 25 January 2019).
19. Micreos-Food-Safety-BV. Phageguard listex application on (food contact) surface areas. Available online: <http://www.phageguard.com/wp-content/uploads/2018/09/PhageGuard-Listex-on-food-contact-surface-areas-EU.pdf> (accessed on 25 January 2019).
20. Micreos-Food-Safety-BV. Phageguard s reduces *Salmonella* on fresh poultry. Available online: <http://www.phageguard.com/wp-content/uploads/2018/03/PhageGuard-ADS-Post-Harvest-Poultry-v82.pdf> (accessed on 25 January 2019).
21. Micreos-Food-Safety-BV. Phageguard e reduces *E. Coli* on leafy green vegetables. Available online: <http://www.phageguard.com/wp-content/uploads/2018/09/PhageGuard-E-ADS-on-leafy-greens.pdf> (accessed on 25 January 2019).
22. Micreos-Food-Safety-BV. Phageguard e reduces e. Coli on beef carcass, parts and trim. Available online: <http://www.phageguard.com/wp-content/uploads/2018/09/PhageGuard-E-ADS-beef.pdf> (accessed on 25 January 2019).
23. Phagelux-Inc. Gras notification: Salmopro®. Available online: <http://www.fda.gov/downloads/food/ingredientspackaginglabeling/gras/noticeinventory/ucm476554.pdf> (accessed on 25 January 2019).
24. Passport-Food-Safety-Solutions-Inc. Finalyse® overhead spray system. Available online: <http://www.passportfoodsafety.com/assets/pdf/Finalyse Product Insert_2017.03.28_LR.pdf> (accessed on 13 February 2019).
25. Passport-Food-Safety-Solutions-Inc. Finalyse®: A novel pre-harvest hide wash. Available online: <http://www.passportfoodsafety.com/assets/pdf/Finalyse Detailer_2017.03.28_LR.pdf> (accessed on 13 February 2019).
26. OmniLytics. Agriphage. Available online: <http://www.agriphage.com/wp-content/uploads/2018/11/67986-1-EPA-Final-Print-Label-AGRIPHAGE-10-18-18.pdf> (accessed on 25 January 2019)
27. OmniLytics. Agriphage-cmm. Available online: <http://www.agriphage.com/wp-content/uploads/2018/11/67986-6-EPA-Final-Print-Label-AGRIPHAGE-CMM-10-23-18.pdf> (accessed on 25 January 2019)
28. OmniLytics. Agriphage-fire blight. Available online: <http://www.agriphage.com/wp-content/uploads/2018/11/EPA-Stamped-Label-67986-8-20180927.pdf> (accessed on 25 January 2019)
29. OmniLytics. Agriphage-citrus canker. Available online: <http://www.agriphage.com/wp-content/uploads/2018/11/EPA-Stamped-Label-67986-9-20180927.pdf> (accessed on 25 January 2019)
30. Phagelux-Inc. Available online: Lexia. <http://www.phagelux.com/en/single/menu_84.htm?menuid=84> (accessed on 2 April 2019).
31. Sharma, M. Lytic bacteriophages: Potential interventions against enteric bacterial pathogens on produce. *Bacteriophage* **2013**, *3*, e25518-e25518.
32. Enviroinvest-Környezetvédelmi-és-Biotechnológiai-Zrt. Erwiphage plus. Available online: <http://www.erwiphage.com> (accessed on 13 February 2019).
33. APS-Biocontrol-Ltd. Biolyse®-pb. Available online: http://www.apsbiocontrol.com/products **2019**.
34. FINK-TEC-GmbH. *Escherichia coli*-specific phage preparation. Available online: <http://www.fda.gov/downloads/Food/IngredientsPackagingLabeling/GRAS/NoticeInventory/UCM597733.pdf> (accessed on 13 February 2019).
35. Proteon-Pharmaceuticals. Bafador®. Available online: <http://www.proteonpharma.com/products/bafador-aquaculture/> (accessed on 13 February 2019).
36. Proteon-Pharmaceuticals. Bafasal®. Available online: <http://www.proteonpharma.com/products/bafasal-poultry/> (accessed on 13 February 2019).
37. Brimrose-Technology-Corporation. Prevention&treatment-bacteriophage product. Available online: <http://www.brimrosetechnology.com/prevention-treatment> (accessed on 13 February 2019).
38. Eliava-Pharmacy. Pyo bacteriophage. Available online: <http://bacteriophagepharmacy.com/product/pyo-bacteriophage/> (accessed on 13 February 2019).
39. Eliava-Pharmacy. Intesti bacteriophage. Available online: <http://bacteriophagepharmacy.com/product/intesti-bacteriophage/> (accessed on 13 February 2019).
40. Eliava-Pharmacy. Ses bacteriophage. **2019**.
41. Eliava-Pharmacy. Enko bacteriophage. Available online: <http://bacteriophagepharmacy.com/product/enko-bacteriophage/> (accessed on 13 February 2019).
42. Eliava-Pharmacy. Fersis bacteriophage. Available online: <http://bacteriophagepharmacy.com/product/fersis-bacteriophage/> (accessed on 13 February 2019).
43. Eliava-Pharmacy. Staphylococcal bacteriophage. Available online: <http://bacteriophagepharmacy.com/product/staphylococcal-bacteriophage/> (accessed on 13 February 2019).
44. Mai, V.; Ukhanova, M.; Visone, L.; Abuladze, T.; Sulakvelidze, A. Bacteriophage administration reduces the concentration of *Listeria monocytogenes* in the gastrointestinal tract and its translocation to spleen and liver in experimentally infected mice. *Int. J. Microbiol.* **2010**, *2010*, 624234.
45. Magnone, J.P.; Marek, P.J.; Sulakvelidze, A.; Senecal, A.G. Additive approach for inactivation of *Escherichia coli* O157:H7, *Salmonella*, and *Shigella spp*. On contaminated fresh fruits and vegetables using bacteriophage cocktail and produce wash. *J. Food Prot.* **2013**, *76*, 1336-1341.
46. Albino, L.A.A.; Rostagno, M.H.; Húngaro, H.M.; Mendonça, R.C.S. Isolation, characterization, and application of bacteriophages for *Salmonella spp*. Biocontrol in pigs. *Foodborne Pathog. Dis.* **2014**, *11*, 602-609.
47. Zhang, H.; Wang, R.; Bao, H. Phage inactivation of foodborne shigella on ready-to-eat spiced chicken. *Poultry Sci.* **2013**, *92*, 211-217.
48. Gras notification of the bacteriophage cocktail: Shigashield tm. Available online: <http://www.fda.gov/downloads/food/ingredientspackaginglabeling/gras/noticeinventory/ucm529756.pdf> (accessed on 25 January 2019).
49. Boyd, E.F.; Brüssow, H. Common themes among bacteriophage-encoded virulence factors and diversity among the bacteriophages involved. *Trends Microbiol.* **2002**, *10*, 521-529.
50. Micreos-Food-Safety-BV. Salmonelextm notification. Available online: <http://www.fda.gov/downloads/Food/IngredientsPackagingLabeling/GRAS/NoticeInventory/ucm505161.pdf> (accessed on 25 January 2019).
51. Alagappan, K.M.; Deivasigamani, B.; Somasundaram, S.T.; Kumaran, S. Occurrence of *Vibrio parahaemolyticus* and its specific phages from shrimp ponds in east coast of india. *Curr. Microbiol.* **2010**, *61*, 235-240.
52. Letchumanan, V.; Chan, K.-G.; Pusparajah, P.; Saokaew, S.; Duangjai, A.; Goh, B.-H.; Ab Mutalib, N.-S.; Lee, L.-H. Insights into bacteriophage application in controlling *Vibrio* species. *Front. Microbiol.* **2016**, *7*.
53. Balogh, **B.**; Jone, J.B. Improved efficacy of newly formulated bacteriophages for management of bacterial spot on tomato. *Plant Dis.* **2003**, *87*, 6.
54. Lee, C.-N.; Lin, J.-W.; Weng, S.-F.; Tseng, Y.-H. Genomic characterization of the intron-containing t7-like phage phil7 of *Xanthomonas campestris†*. *Appl. Environ. Microb.* **2009**, *75*, 7828.
55. Yu, J.G.; Lim, J.A.; Song, Y.R.; Heu, S.; Kim, G.H.; Koh, Y.J.; Oh, C.S. Isolation and characterization of bacteriophages against *Pseudomonas syringae* pv. Actinidiae causing bacterial canker disease in kiwifruit. *J. Microbiol. Biotechn.* **2016**, *26*, 385-393.
56. Wittmann, J.; Gartemann, K.-H.; Eichenlaub, R.; Dreiseikelmann, B. Genomic and molecular analysis of phage cmp1 from *Clavibacter michiganensis* subspecies *michiganensis*. *Bacteriophage* **2011**, *1*, 6-14.
57. Gill, J.J.; Svircev, A.M.; Smith, R.; Castle, A.J. Bacteriophages of *Erwinia amylovora*. *Appl. Environ. Microb.* **2003**, *69*, 2133.
58. Born, Y.; Fieseler, L.; Marazzi, J.; Lurz, R.; Duffy, B.; Loessner, M.J. Novel virulent and broad-host-range *Erwinia amylovora* bacteriophages reveal a high degree of mosaicism and a relationship to enterobacteriaceae phages. *Appl. Environ. Microb.* **2011**, *77*, 5945.
59. Ahmad, A.A.; Ogawa, M.; Kawasaki, T.; Fujie, M.; Yamada, T. Characterization of bacteriophages cp1 and cp2, the strain-typing agents for *Xanthomonas axonopodis* pv. Citri. *Appl. Environ. Microb.* **2014**, *80*, 77.
60. Meczker, K.; Domotor, D.; Vass, J.; Rakhely, G.; Schneider, G.; Kovacs, T. The genome of the *Erwinia amylovora* phage phieah1 reveals greater diversity and broadens the applicability of phages for the treatment of fire blight. *FEMS Microbiol. Lett.* **2014**, *350*, 25-27.
61. Adriaenssens, E.M.; Van Vaerenbergh, J.; Vandenheuvel, D.; Dunon, V.; Ceyssens, P.-J.; De Proft, M.; Kropinski, A.M.; Noben, J.-P.; Maes, M.; Lavigne, R. T4-related bacteriophage limestone isolates for the control of soft rot on potato caused by ‘dickeya solani’. *PLOS ONE* **2012**, *7*, e33227.
62. O'Sullivan, L.; Bolton, D.; McAuliffe, O.; Coffey, A. Bacteriophages in food applications: From foe to friend. *Annu. Rev. Food Sci. Technol.* **2019**.
63. Pasternack, G.; Sulakelidze, A. Novel shigella bacteriophages and uses thereof. *Intralytix, Inc., Baltimore, MD (US)* **2016**.
64. D'Souza, D.H.; Su, X. Efficacy of chemical treatments against murine norovirus, feline calicivirus, and ms2 bacteriophage. *Foodborne Pathog. Dis.* **2010**, *7*, 319-326.
65. Kobayashi, H.; Tsuzuki, M.; Koshimizu, K.; Toyama, H.; Yoshihara, N.; Shikata, T.; Abe, K.; Mizuno, K.; Otomo, N.; Oda, T. Susceptibility of hepatitis b virus to disinfectants or heat. *J. Clin. Microbiol.* **1984**, *20*, 214-216.
66. Maillard, J.Y.; Beggs, T.S.; Day, M.J.; Hudson, R.A.; Russell, A.D. Effect of biocides on ms2 and k coliphages. *Appl. Environ. Microb.* **1994**, *60*, 2205-2206.
67. Cook, A.M.; Brown, W.R.L. Inactivation of a bacteriophage by chemical antibacterial agents. *J. Pharm. Pharmacol.* **1964**, *16*, 611-617.
68. Smith, H.W.; Huggins, M.B.; Shaw, K.M. Factors influencing the survival and multiplication of bacteriophages in calves and in their environment. *J. Gen. Microbiol.* **1987**, *133*, 1127-1135.
69. Brown, B.L.; Fuerst, R. Ethylene oxide sterilization of tissue culture media. *Science (New York, N.Y.)* **1963**, *142*, 1654-1655.
70. Thurston-Enriquez, J.A.; Haas, C.N.; Jacangelo, J.; Gerba, C.P. Chlorine inactivation of adenovirus type 40 and feline calicivirus. *Appl. Environ. Microb.* **2003**, *69*, 3979-3985.
71. Zyara, A.M.; Torvinen, E.; Veijalainen, A.M.; Heinonen-Tanski, H. The effect of chlorine and combined chlorine/uv treatment on coliphages in drinking water disinfection. *J. Water Health* **2016**, *14*, 640-649.
72. Campagna, C.; Villion, M.; Labrie, S.J.; Duchaine, C.; Moineau, S. Inactivation of dairy bacteriophages by commercial sanitizers and disinfectants. *Int. J. Food Microbiol.* **2014**, *171*, 41-47.
73. Grunert, A.; Frohnert, A.; Selinka, H.C.; Szewzyk, R. A new approach to testing the efficacy of drinking water disinfectants. *Int. J. Hyg. Environ. Health* **2018**, *221*, 1124-1132.
74. Binetti, A.G.; Reinheimer, J.A. Thermal and chemical inactivation of indigenous *Streptococcus thermophilus* bacteriophages isolated from argentinian dairy plants. *J. Food Prot.* **2000**, *63*, 509-515.
75. Ebrecht, A.C.; Guglielmotti, D.M.; Tremmel, G.; Reinheimer, J.A.; Suarez, V.B. Temperate and virulent *Lactobacillus delbrueckii* bacteriophages: Comparison of their thermal and chemical resistance. *Food Microbiol.* **2010**, *27*, 515-520.
76. Briggiler Marco, M.; De Antoni, G.L.; Reinheimer, J.A.; Quiberoni, A. Thermal, chemical, and photocatalytic inactivation of *Lactobacillus plantarum* bacteriophages. *J. Food Prot.* **2009**, *72*, 1012-1019.
77. Dunkin, N.; Weng, S.; Schwab, K.J.; McQuarrie, J.; Bell, K.; Jacangelo, J.G. Comparative inactivation of murine norovirus and ms2 bacteriophage by peracetic acid and monochloramine in municipal secondary wastewater effluent. *Environ. Sci. Technol.* **2017**, *51*, 2972-2981.
78. Hayes, S.; Murphy, J.; Mahony, J.; Lugli, G.A.; Ventura, M.; Noben, J.-P.; Franz, C.M.A.P.; Neve, H.; Nauta, A.; Van Sinderen, D. Biocidal inactivation of *Lactococcus lactis* bacteriophages: Efficacy and targets of commonly used sanitizers. *Front. Microbiol.* **2017**, *8*, 107-107.
79. Agun, S.; Fernandez, L.; Gonzalez-Menendez, E.; Martinez, B.; Rodriguez, A.; Garcia, P. Study of the interactions between bacteriophage phiipla-rodi and four chemical disinfectants for the elimination of *Staphylococcus aureus* contamination. *Viruses* **2018**, *10*.
80. Morin, T.; Martin, H.; Soumet, C.; Fresnel, R.; Lamaudiere, S.; Le Sauvage, A.L.; Deleurme, K.; Maris, P. Comparison of the virucidal efficacy of peracetic acid, potassium monopersulphate and sodium hypochlorite on bacteriophages p001 and ms2. *J. Appl. Microbiol.* **2015**, *119*, 655-665.
81. Tomat, D.; Balagué, C.; Aquili, V.; Verdini, R.; Quiberoni, A. Resistance of phages lytic to pathogenic *Escherichia coli* to sanitisers used by the food industry and in home settings. *Food Sci. Technol. Int.* **2018**, *53*, 533-540.
82. Mercanti, D.J.; Guglielmotti, D.M.; Patrignani, F.; Reinheimer, J.A.; Quiberoni, A. Resistance of two temperate *Lactobacillus paracasei* bacteriophages to high pressure homogenization, thermal treatments and chemical biocides of industrial application. *Food Microbiol.* **2012**, *29*, 99-104.
83. Branston, S.D.; Stanley, E.C.; Ward, J.M.; Keshavarz-Moore, E. Determination of the survival of bacteriophage m13 from chemical and physical challenges to assist in its sustainable bioprocessing. *Biotechnol. Bioproc. E.* **2013**, *18*, 560-566.
84. Sands, J.A. Inactivation and inhibition of replication of the enveloped bacteriophage phi6 by fatty acids. *Antimicrob. Agents Chemother.* **1977**, *12*, 523-528.
85. Hirotani, H.; Ohigashi, H.; Kobayashi, M.; Koshimizu, K.; Takahashi, E. Inactivation of t5 phage by cis‐vaccenic acid, an antivirus substance from *Rhodopseudomonas capsulata*, and by unsaturated fatty acids and related alcohols. *FEMS Microbiol. Lett.* **1991**, *77*, 13-18.
86. Reinhardt, A.; Cadden, S.; Sands, J.A. Inhibitory effect of fatty acids on the entry of the lipid-containing bacteriophage pr4 into *Escherichia coli*. *J. Virol.* **1978**, *25*, 479-485.
87. Allwood, P.B.; Malik, Y.S.; Hedberg, C.W.; Goyal, S.M. Effect of temperature and sanitizers on the survival of feline calicivirus, *Escherichia coli*, and f-specific coliphage ms2 on leafy salad vegetables. *J. Food Prot.* **2004**, *67*, 1451-1456.
88. Sommer, J.; Fister, S.; Gundolf, T.; Bromberger, B.; Mester, P.-J.; Witte, A.; Kalb, R.; Rossmanith, P. Virucidal or not virucidal? That is the question—predictability of ionic liquid’s virucidal potential in biological test systems. *Int. J. Mol. Sci.* **2018**, *19*, 790.
89. Fister, S.; Mester, P.; Sommer, J.; Witte, A.K.; Kalb, R.; Wagner, M.; Rossmanith, P. Virucidal influence of ionic liquids on phages p100 and ms2. *Front. Microbiol.* **2017**, *8*.
90. Byrne, N.; Rodoni, B.; Constable, F.; Varghese, S.; Davis, J.H. Enhanced stabilization of the tobacco mosaic virus using protic ionic liquids. *Phys. Chem. Chem. Phys.* **2012**, *14*, 10119-10121.

| 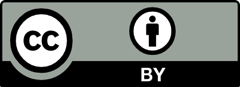 | © 2019 by the authors. Submitted for possible open access publication under the terms and conditions of the Creative Commons Attribution (CC BY) license (http://creativecommons.org/licenses/by/4.0/). |
| --- | --- |
