# Supplementary material for: Don’t Shut the Stable Door after the Phage Has Bolted—The Importance of Bacteriophage Inactivation in Food Environments
Source: Viruses. 2019 May 22;11(5):468. doi: 10.3390/v11050468 (PMC6563225; doi:10.3390/v11050468)
Supplement: Supplementary file 1 [file viruses-11-00468-s001.zip › viruses-493168 final supplementary/viruses-493168 supplementary.pdf]

viruses

MDPI

Review

Don’t Shut the Stable Door after the Phage has Bolted—the Importance of Bacteriophage Inactivation in Food Environments

Julia Sommer [1], Christoph Trautner [1], Anna Kristina Witte [1,3], Susanne Fister [4], Dagmar Schoder [2], Peter Rossmanith [1,2] and Patrick-Julian Mester [1,*]

[1] Christian Doppler Laboratory for Monitoring of Microbial Contaminants, Department for Farm Animal and Public Health in Veterinary Medicine, University of Veterinary Medicine, Veterinaerplatz 1, 1210 Vienna, Austria; julia.sommer@vetmeduni.ac.at (J.S.); christoph.trautner@vetmeduni.ac.at (C.T.); anna.witte@vetmeduni.ac.at (A.K.W.); peter.rossmanith@vetmeduni.ac.at (P.R.)

[2] Unit of Food Microbiology, Institute of Food Safety, Food Technology and Veterinary Public Health, Department for Farm Animal and Public Health in Veterinary Medicine, University of Veterinary Medicine, Veterinaerplatz 1, 1210 Vienna, Austria; dagmar.schoder@vetmeduni.ac.at

[3] Current address: HTK Hygiene Technologie Kompetenzzentrum GmbH, Buger Str. 80, 96049 Bamberg, Germany

[4] Former member of Christian Doppler Laboratory for Monitoring of Microbial Contaminants; susanne.fister1@gmail.com

* Correspondence: patrick-julian.mester@vetmeduni.ac.at; Tel: +43-25077-3529; Fax: +43-25077-3590

viruses MDPI

Table S1. List of currently commercial available bacteriophage product used in the fields of agriculture, animal rearing and treatment and food and feed sector and their suggested application strategies.

Company	Phage Product	Application					References
		Usage on	Application type	Concentration	Time intervals	Field of application	
Intralytix, Inc. (Baltimore, MD, USA)	ListShield™	For direct food applications	Typically applied directly on food surfaces by spraying at a food product surface	Concentration of approximately 1 - 2 mL per 250 sq. cm	/	Post-Harvest - Food Processing Facility	[1]
	EcoShield™	For direct food applications	Industrial sprayer for large direct food applications; handheld spryer for smaller bench-scale food applications	Diluted working solution - a concentration of 1 - 4 mL per pound of food product	/	Post-Harvest - Food Processing Facility	[2]
	SalmoFresh™	For direct food applications - Red meat and poultry	Industrial sprayer for large direct food applications; handheld spryer for smaller bench-scale food applications	Diluted working solution - a concentration of 1 - 4 mL per pound of food product	/	Post-Harvest - Food Processing Facility sing Facility	[3]
	ShigaShield™ (ShigaActive™)	Ready-to-eat meats, fish and shellfish, and fresh and processed fruits and vegetables, and dairy products	Direct application onto products	/	/	Post-Harvest - Food Processing Facility	[4]
	ListPhage™	/	/	/	/	Post-Harvest	[5,6]

						– Pet Food Safety	
	SalmoLyse®	/	/	/	/	Post-Harvest – Pet Food Safety	[7,8]
	Ecolicide®	/	/	/	/	Post-Harvest – Pet Food Safety	[9-11]
	Ecolicide PX™	/	/	/	/	Pre-Harvest Interventions	[9-11]
	PLSV-1™	/	/	/	/	Pre-Harvest veterinary applications	[12]
	INT-401™	/	/	/	/	Pre-Harvest veterinary applications	[12,13]
Micreos Food Safety (Wageningen, Netherlands)	PhageGuard Listex™	Surface ripened washed rinds and white mould cheeses; ripening culture to protect the smearing robot against cross contamination during brushing and washing	Application of PhageGuard Listex is either by spraying (1ml per 100 cm2) or by immersion and is effective in solution	0.5% ($1x10^8$) - 1% ($2x10^9$); in pasteurized milk or on the rind ,.. etc.	/	Post-Harvest - Food Processing Facility	[14]

	PhageGuard Listex™	Whole fish, filets and in the environment	Spray onto product prior to packaging; Spray into package ; Spray onto slicer blade/dicer blades ; Dipping/immersion into a solution containing Listex	2% (4x10⁹)	/	Post-Harvest - Food Processing Facility	[15,16]
	PhageGuard Listex™	Meat products, RTE , frozen vegetables; products; meat slicer	Spray onto product prior to packaging; Spray into package ; Spray onto slicer blade/dicer blades ; Dipping/immersion into a solution containing Listex	1% (2x10⁹)	/	Post-Harvest - Food Processing Facility	[17,18]
	PhageGuard Listex™	Food-contact surfaces	Spray on the surface, add so much that the surface becomes wet	1% dilution	Daily	Post-Harvest - Food Processing Facility	[19]
	PhageGuard Listex™	Biofilms/Hot Spots treatments	Spray on the surface, add so much that the surface becomes wet	5% dilution	Daily, until test negative	Post-Harvest - Food Processing Facility	[19]
	PhageGuard Listex™	Food-processing	Spray on the surface, add so much that the	1% dilution	Weekly/Bi-	Post-Harvest -	[16,19]

		environments	surface becomes wet		weekly	Food Processing Facility	
	PhageGuard S™	Poultry	On line dipping / immersion / Spray; Spray into a final package or Spray onto product prior to grinding	1% dilution	/	Post-Harvest - Food Processing Facility	[20]
	PhageGuard E™	Leafy green Vegetables	Using conventional spry or electrostatic spry or dipping	$3x10^7$ - $3x10^8$ PFU/cm^2	/	Post-Harvest - Food Processing Facility	[21]
	PhageGuard E™	Beef carcass, parts and trim	Spray or Dip application	$3x10^7$ - $3x10^8$ PFU/cm^2	/	Post-Harvest - Food Processing Facility	[22]
	Salmonelex™	Pork and poultry products	/	levels up to I 0^8 PFU/g offood	/		[23]
Passport Food Safety Solutions (West Des Moines, IA, USA)	Finalyse®	Cattle	Pre-harvest Hide Wash	0.22% solution applied as an overhead spray system in the holding pens or lairage area spray system	Application timeline typically 1 to 1 ½ minutes	Pre-Harvest	[24,25]
OmniLytics (Salt Lake City) Phagelux (Shanghai, China)	Agriphage™	Tomato & Pepper	Greenhouse Seedling Treatment[1]	1 pint of AgriPhage per 50-100 gallons of water per 9600 square feet of greenhouse space	Apply daily treatments to foliage by spraying or	Agriculture - Pre-Harvest	[26]

Phagelux (Montreal, Canada)					fogging		
		Tomato & Pepper	Field Treatment – Ground Application[1]	1 to 2 pints of AgriPhage per 50-100 gallons of water per acre	Repeat application 1-3 times per week	Agriculture - Pre-Harvest	[26]
	Agriphage™ & Agriphage™ CMM	Tomato & Pepper	Field Treatment – Aerial Application	1-2 pints per acre in a minimum of 5 gallons of water per acre	/	Agriculture - Pre-Harvest	[26,27]
	Agriphage™ CMM	Tomato	Greenhouse Seedling Treatment[1]	1 pint of AgriPhage-CMM per 12-25 gallons of water per 9600 square feet of greenhouse space	Apply daily treatments to foliage by spraying or fogging	Agriculture - Pre-Harvest	[27]
		Tomato	Greenhouse Treatment – For Tomatoes Grown Hydroponically[1]	to 1 quart of AgriPhage-CMM per 12-25 gallons of water per 9600 square feet of greenhouse space	Apply to foliage by spraying or fogging. Repeat application 1-3 times per week	Agriculture - Pre-Harvest	[27]
		Tomato	Field Treatment – Ground Application[1]	1 to 2 pints of AgriPhage-CMM per 50-100 gallons of water per acre	Repeat application 1-3 times per week	Agriculture - Pre-Harvest	[27]
	Agriphage™ FireBligth	Apples & Pears	Field Treatment – Ground Application[1]	1 to 2 quarts of AGRIPHAGEFIRE BLIGHT per 50-100 gallons of	Repeat application weekly or as needed.	Agriculture - Pre-Harvest	[28]

				water per acre.			
	Agriphage™ CitrusCranker	Citrus e.g., orange, grapefruit, pummelo, lemon, lime, tangerine, tangelo, or kumquat	Field Treatment – Ground Application[1]	1 to 2 quarts of AGRIPHAGECITRUS CANKER per 50-100 gallons of water per acre	Repeat application weekly or as needed	Agriculture - Pre-Harvest	[29]
	Lexia	*Penaeus vannamei* shrimp	„In water" -Treatment	/	/	Aqua-culture	[30]
	SalmoPro® (2015)	Food	/	Application rate at a maximum of 1 x 10^8 PFU/g of food	/	Post-Harvest - Food Processing Facility	[23]
	SalmoPro® (2018)	Poultry, Red meat, Fruit, Vegetables, Eggs, Fish and shellfish	/	Application of SalmoPro® at a maximum rate of $1x10^8$ PFU/g of food	/	Post-Harvest - Food Processing Facility	[4]
	Armament™	Poultry and cattle	Application as a spray mist or wash on the feathers of live poultry prior to slaughter to decrease pathogen transfer to meat	/	/	Pre-Harvest	[23,31]
Enviroinvest Környezetvédelmi és Biotechnológiai Zrt.	Erwiphage PLUS	Apple	Application as a spray	/	During flowering periode	Agriculture - Pre-Harvest	[32]

APS Biocontrol Ltd	Biolyse®-PB	Potatoes	Application via spray-bar equipment	/	/	Post-Harvest - Food Processing Facility	[33]
CheilJedang Corporation	Biotector® S1	Animal feed	Replace antibiotics in animal feed	/	/	Pre-Harvest - Animal Breeding	[23]
	Biotector® S4	Animal feed	Replace antibiotics in animal feed	/	/	Pre-Harvest - Animal Breeding	[23]
FINK TEC GmbH (Hamm, Germany)	Secure Shield E1	Meat processing facilities; Beef carcasses	Spray systems in meat processing facilities	Approximately $1.5x10^{11}$ phage particles per carcass	/	Post-Harvest - Food Processing Facility	[34]
Proteon Pharmaceuticals	BAFADOR®	Commercial aquaculture, fishes	„In water" -Treatment	/	/	Pre-Harvest - Animal Breeding	[35]
	BAFASAL®	Poultry	Feed additive that eliminates, or prevents, salmonella infection in the digestive tract in poultry	Applied by being added to the drinking water	/	Pre-Harvest - Animal Breeding	[36]
Brimrose Technology Corporation	Pyo Bacteriophage	/	Used in the treatment and prophylaxis of purulent inflammatory and enteric infectious diseases	/	/	Post-Harvest – Pet Food Safety	[37,38]
	Intesti	/	Can be used in	/	/	Post-Harvest	[37,39]

viruses MDPI

	Bacteriophage		therapeutic and prophylactic purposes in pyo-inflammatory and enteric infections			– Pet Food Safety	
	SES Bacteriophage	/	Can be used in therapeutic and prophylactic purposes in pyo-inflammatory and enteric infections	/	/	Post-Harvest – Pet Food Safety	[37,40]
	EnkoPhagum	/	Can be used in therapeutic and prophylactic purposes in pyo-inflammatory and enteric infections	/	/	Post-Harvest – Pet Food Safety	[37,41]
	Fersisi Bacteriophage	/	Can be used in therapeutic and prophylactic purposes in pyo-inflammatory and enteric infections	/	/	Post-Harvest – Pet Food Safety	[37,42]
	Mono-phage Preparations	/	/	/	/	Post-Harvest – Pet Food Safety	[37]
	Staphylococcal Bacteriophage	/	Used in the treatment and prophylaxis of infections	/	/	/	[43]

[1] AgriPhage may be applied up to and including the day of harvest.

Table S2. List of currently commercial available bacteriophage product used in the fields of agriculture, animal rearing and treatment and food and feed sector.

Company	Phage Product	Target Organisms	Used Phages	Taxonomy	Application	Reference
Intralytix, Inc. (Baltimore, MD, USA)	ListShield™	*Listeria monocytogenes*	Six-phages cocktail	Caudovirales	Food Safety	[44]
	EcoShield™	*Escherichia coli* O157:H7	Three-phages cocktail	Caudovirales	Food Safety	[45]
	SalmoFresh™	pathogenic Salmonella-Serotypes; *Salmonella spp.*	Six-phages cocktail;	Caudovirales	Food Safety	[45,46]
	ShigaShield™ (ShigaActive™)	*Shigella spp.*	Five-phage cocktail; SHFML-11, SHFML-26, SHSML-45, SHBML-50-1, SHSML-52-1	Caudovirales: Myoviridae	Food Safety	[45,47,48]
	ListPhage™	*Listeria monocytogenes*	/	Caudovirales	Pet Food Safety	
	SalmoLyse®	*Salmonella*	/	Caudovirales	Pet Food Safety	
	Ecolicide®	*Escherichia coli* O157:H7	/	Caudovirales	Pet Food Safety	
	Ecolicide PX™	*Escherichia coli* O157:H7	/	Caudovirales	Pre-Harvest Interventions	
	PLSV-1™	*Salmonella*	/	/	Phage preparations for veterinary applications	[12]
	INT-401™	*Clostridium perfringens*	Five bacteriophages (CPAS-7, CPAS-12, CPAS-15, CPAS-16 and CPLV-42)	Caudovirales: Myoviridae, Siphoviridae	Phage preparations for veterinary applications	[12,13]
Micreos Food Safety (Wageningen,	PhageGuard Listex™	*Listeria monocytogenes*	Single phage; P100	Caudovirales: Myoviridae	Food Safety	[14,15,17]

Netherlands)	PhageGuard S™	*Salmonella spp.*	/	Caudovirales	Food Safety	[20,46]
	PhageGuard E™	*Escherichia coli* O157:H7	/	Caudovirales	Food Safety	[21,22,49]
	SalmonelexTM	*Salmonella* serovars	S16 and FO1a	Caudovirales: Myoviridae	Food Safety	[23,50]
Passport Food Safety Solutions (West Des Moines, IA, USA)	Finalyse®	*Escherichia coli* O157:H7	/	Caudovirales	Pre-Harvest hide wash	[24,25,49]
	Lexia	*Vibrio parahemolyticus*	Three-phages cocktail: VP7, VP46, VP48	Caudovirales: Myoviridae	Aquaculture	[30,51,52]
OmniLytics (Salt Lake City)<br><br>Phagelux (Shanghai, China)<br><br>Phagelux (Montreal, Canada)	Agriphage™	*Xanthomonas campestris* pv. *vesicatoria* and *Pseudomonas syringe* pv. *tomato*	Phage phil7; 6-8 phages	Caudovirales: Myoviridae	Agriculture	[26,53-55]
	Agriphage™ CMM	*Clavibacter michiganensis* subsp. *michiganensis*	/	Caudovirales: Mycobacteriophage	Agriculture	[27,56]
	Agriphage™ FireBligth	*Erwinia amylovora*	/	Caudovirales	Agriculture	[28,57,58]
	Agriphage™ CitrusCranker	*Xanthomonas citri* subsp. *citri*	/	Caudovirales	Agriculture	[29,49,59]
	SalmoPro® (2015)	*Salmonella enterica*	Two-phage cocktail: BP-63 and BP-12	Caudovirales: Myoviridae, Siphoviridae, Podoviridae	Food Safety	[23]
	SalmoPro® (2018)	*Salmonella enterica*	Two-phage cocktail: BP-63 and LVR16-A	Caudovirales: Myoviridae, Siphoviridae	Food Safety	[4]
	Armament™	*Salmonella spp.*	/	/	Food Safety	[23,31]

Enviroinvest Környezetvédelmi és Biotechnológiai Zrt.	Erwiphage PLUS	*Erwinia amylovora*	PhiEaH2, PhiEaH1	Caudovirales: Siphoviridae	Agriculture	[32,57,58,60]
APS Biocontrol Ltd	Biolyse®-PB	Specific against soft rot Entero-bacteriacea	/	Caudovirales: Myoviridae	Agriculture; Food Safety	[33,61]
CheilJedang Corporation	Biotector® S1	*Salmonella Galliinarum* (SG) and *S. Pullorum* (SP)	/	/	Pet Food Safety	[23]
	Biotector® S4	*Salmonella Galliinarum* (SG) and *S. Pullorum* (SP)	/	/	Pet Food Safety	[23]
FINK TEC GmbH (Hamm, Germany)	Secure Shield E1	*E. coli* & *E. coli* O157:H7	(AB27), (TB49), (TB120), (KRA2), (TB69), (BO1), (EW2), (TB6A), (GWF), (HAM53), (MP57), (TB11)	Caudovirales: Myoviridae, Podoviridae	Food Safety	[34]
Proteon Pharmaceuticals	BAFADOR®	*Pseudomonas* and *Aeromonas*	/	/	Pre-Harvest Interventions Aquaculture	[35,62]
	BAFASAL®	*Salmonella*	/	/	Pre-Harvest Interventions	[36]
Brimrose Technology Corporation / Eliava Authorized Pharmacy	Pyo Bacteriophage	Staphylococcus (*S. aureus*), Streptococcus (*S. pyogenes*, *S. sanguis*, *S. salivarius*, *S. agalactiae*), *E.coli* (Different types), *Pseudomonas aeruginosa*, *Proteus mirabilis* and *P. vulgaris*	5 components/phages	/	Pet Food Safety	[37,38]
	Intesti Bacteriophage	*Shigella flexneri* (Serotypes 1,2,3,4; *Shigella sonnei*; *Shigella newcastle*;	17 components/phages	/	Pet Food Safety	[37,39]

		Salmonella paratyphi A and *Salmonella paratyphi B*; *Salmonella typhimurium*; *Salmonella enteritidis*; *Salmonella choleraesuis*; *Salmonella oranienburg*; *Escherichia coli*; *Proteus vulgaris*, *P. mirabilis*; *Staphylococcus aureus*; *Pseudomonas aeruginosa*; *Enterococcus faecalis*				
	SES Bacteriophage	Staphylococcus (*S. aureus*, S. *epidermidis*), Streptococcus (*S. pyogenes*, *S. sanguis*, *S. salivarius*, *S. agalactiae*); different types of *E.coli*	3 components/phages	/	Pet Food Safety	[37,40]
	EnkoPhagum	*Salmonellae* [Paratyphus A, Paratyphus B, Typhimurium, Enteritidus, Choleraesuis, Oranienburg, Dublin, Anatum], *Shigellas* [Flexner, Zonne], Enterepathogenic serotypes of *Escherichia coli* [10 types], *Staphylococcus* [3 types])	23 components/phages	/	Pet Food Safety	[37,41]
	Fersisi Bacteriophage	Staphylococcus (*S. aureus*, *S. epidermidis*) Streptococcus (*S. pyogenes*, *S. sanguis*, *S. salivarius*, *S. agalactiae*)	7 components/phages	/	Pet Food Safety	[37,42]
	Mono-phage Preparations	Staphylococcal, *E. coli*, Streptococcal, *Pseudomonas aeruginosa*, Proteus	5 components/phages	/	Pet Food Safety	[37]
Eliava Authorized Pharmacy	Staphylococcal Bacteriophage	*Staphylococcus aureus*	/	/	/	[43]

7

Table S3. List of patents of bacteriophages submitted by commercial phage product manufacturers for usage in food environments.

Company	Patent	Target Organisms	Used Phages	Taxonomy	Application	Reference
Intralytix, Inc. (Baltimore, MD, USA)	Patent No.: US 8,685,697 B1	*Listeria monocytogenes*	Patent 2014: LMSP-25, LMTA-34, LMTA-57, LMTA-94, or LMTA-148;	Caudovirales	Food Safety	[5]
	Patent No.: US 7,507,571 B2	*Listeria monocytogenes*	Patent 2009: List 1, List 2, List3, List 4, List 36 and List38	Caudovirales	Food Safety	[6]
	Patent No.: US 7.625,556 B2	*Escherichia coli* O157:H7	Patent 2009: ECTA-47, ECML-83, ECML-119, and ECML-122;	Caudovirales	Food Safety	[9]
	Patent No.: US 7,635,584 B2	*Escherichia coli* O157:H7	Patent 2009_2: ECML-4	Caudovirales	Food Safety	[10]
	Patent No.: US 7.625,741 B2	*Escherichia coli* O157:H7	Patent 2009_1: ECML-117 and ECML-134	Caudovirales	Food Safety	[11]
	Patent No.: US 7,674.467 B2	pathogenic Salmonella-Serotypes; *Salmonella spp.*	SPT-1, SBA-178, SBA-1781, SIT 128, SSE-121 and SDT-15	Caudovirales	Food Safety	[7]
	Patent No.: US 8,685,696 B2	pathogenic Salmonella-Serotypes; *Salmonella spp.*	STML-198, SNN-387, SEML-239-1, STML-13-1,SKML-39. SEML-24, and STA-202	Caudovirales	Food Safety	[8]
	Patent No.: US 2016/0215273 A1	*Shigella spp.*	SHFML-26, SHFML-11, SHSML-45, SHSML-52-1, SHBML-50-1,	Caudovirales: Myoviridae	Food Safety	[63]

			SHBML-50-2, SHSML-52-2, SHSML-36, and SHFML-21			

viruses

MDPI

Table S4. Status of select biocides with issuing agencies and permitted maximum levels.

Substance class	Substance	Hazards	CAS reg. #	Canada (HC)	U.S. (FDA)	EU (ECHA)	FAO/WHO	References
Aldehydes	Glutaraldehyde	C,A, I, H, E	111-30-8	Not approved	Food additive/ Permitted maximum levels of oxidizing/reducing agent: 250 ppm	Approved, Regulation (EU) 2015/1759	Not approved	[64-66]
	Formaldehyde	F, C, I, H	50-00-0	Not approved	Food additive	Not approved	Not approved	[67,68]
	Ethylene oxide	F, G, A, I, H	75-21-8	Not approved, under regulation by PCPA	Food additive	Not approved	Not approved	[69]
Chlorine and chlorine releasing agents	Chlorine	O, G, A, I, E	7782-50-5	Approved for flour and whole wheat flour only; Permitted maximum levels for chlorine gas:	Flour and whole wheat flour only; Permitted maximum levels for chlorine gas: GMP	Not approved *	Approved for flour only; FAO: 2.5 g/kg (treatment) for flour only<br>WHO: 2.5 g/kg (treatment) for flour only	[70,71]
	Chlorine dioxide	O, G, C, A, I, E	10049-04-4	Approved for flour and whole wheat flour only; Permitted maximum levels: GMP	Food additive; Permitted maximum levels: GMP; antimicrobial wash solutions: 3 ppm sanitizers: 100 ppm (minimum), 200 ppm (maximum)	Under review *	Approved for flour only; Permitted maximum levels: WHO: 30 mg/kg (treatment), 75 mg/kg (conditional) for flour only	[72,73]

	Sodium hypochlorite	C, I, E	7681-52-9	Approved, permitted on starch only; Permitted maximum levels: GMP	Food additive; Permitted maximum levels: GMP except starch residual total chlorine: 0.055 pds per pd dry starch, 0.0082 pds per pd dry starch when used as bleaching agent	Approved, Regulation (EU) 2017/1273; Permitted maximum levels: 10 µg per kg according to Regulation (EC) No 396/2005	Not approved	[72,74-76]
	Monochloramine	C, I, H	10599-90-3	Not approved	Not approved	Approval for drinking water in progress	Not approved	[77]
	Chloramine-T	C, I, H	127-65-1	Not approved	Not approved	Approval in progress	Not approved	[67]
Iodophores	Polyvinlylpyrrolidine-iodine	C, I, E	25655-41-8	Not approved	Not approved	Approved	Not approved	[78]
Peroxides	Hydrogen peroxide	O, C, I	7722-84-1	Food additive; Permitted maximum levels: bleaching agents and starch: GMP liquid whey: 100 ppm brewers' mash: 135 ppm residual	Food additive, GRAS; Permitted maximum levels: bottled water: 23 mg/kg food starch: 0.45 % active residual oxygen from hydrogen peroxide wash water: 59 ppm hydrogen peroxide solution: 35 %	Approved, Regulation (EU) 2015/1730; Permitted maximum levels: according to Regulation (EU) 2017/1777 (Annex IV) for hydrogen peroxide	Approved; Permitted maximum levels: WHO: unspecified amounts of residue from wash solutions are permitted	[78,79]

					21CFR184.1366			
	Potassium peroxymonosulfa te	O,C, I, H	37222-66-5	Not approved	Not approved	Not approved	Not approved	[80]
	Peracetic acid	F, C, A, I, E	79-21-0	Food additive; Permitted maximum levels: for starch: GMP	Food additive; Permitted maximum levels: residual on fruits: 80 ppm antimicrobial agent: 220 ppm	Approved, Regulation (EU) 2016/672	Approved (peroxyacetic acids); Permitted maximum levels: no safety concern due to degradation into compounds of low toxicity	[74-76]
	Peroctanoic acid	F, C, E	33734-57-5	Not approved	Food additive; Permitted maximum levels: poultry carcasses, poultry parts and organs: GMP in sanitizers: restrictions apply depending on the application and type of food contact surface	Approval in progress	Approved	[81,82]
Phenols	Nonylphenols	C, I, E	9016-45-9	not approved	Not approved	Not approved	Not approved	[82]
	5-Chloro-2-(2,4-dichlorophenoxy) phenol (Triclosan)	I, E	3380-34-5	Not approved	Not approved	Not approved	Not approved	[79]
Quarternary ammonium	Benzalkonium chloride	C, I, E	8001-54-5	Not approved	Not approved	Under review, Regulation (EU)	Not approved	[75,78,79]

compounds						1119/2014		
Alcohols	Ethanol	F, I, H	64-17-5	Food additive; Permitted maximum levels: carrier/extractant: GMP	Food additive, GRAS	Approval in progress	Approved; Permitted maximum levels: GMP	[74-76]
	Isopropanol	F, I	67-63-0	Food additive; Permitted maximum levels: carrier/extractant: 50 ppm residual in fish protein: 0.15%	Food additive	Approved, Regulation (EU) 2015/407	Approved; Permitted maximum levels: no safety concern due to low toxicity	[74-76]
Acids	Trisodium phosphate	C, I	7601-54-9	Food additive; Permitted maximum levels: alcoholic beverages: GMP cheeses: 3.5% as anhydrous salt or 4% total anhydrous salt each when in combination with other salts	Food additive, GRAS; Permitted maximum levels: steam: GMP	Not approved	Approved; Permitted maximum levels: FAO: limits for total residual phosphorous: 3.52 g/kg (humectants), 1.32 g/kg (emulsifiers)	[64,75,82]
	Sulfuric acid	C	7664-93-9	Approved; Permitted maximum levels: starch: GMP	Food additive, GRAS; Permitted maximum levels: caramel, starch: GMP in sanitizers: varies with type and	Not approved but preregistered; Permitted maximum levels: not required according to	Approved;	[83]

					concentration of other components	Regulation (EU) No 1146/2014		
	Sodium hypochlorite	C, I, E	7681-52-9	Food additive, permitted on starch only; Permitted maximum levels:starch: GMP	Food additive	Approved, Regulation (EU) 2017/1273	Not approved	[72,74-76]
	Fatty acids	varies	124-07-2 (C8:0), 334-48-5 (C10:0), 143-07-7 (C12:0), 544-63-8 (C14:0), 57-10-3 (C16:0), 57-11-4 (C18:0), 112-80-1 (C18:1), 60-33-3 (C18:2)	Not approved except C18:0; Permitted maximum levels: GMP	Approved: C8:0, C10:0, C12:0, C14:0, C16:0, C18:0 (GRAS), C18:1 (GRAS), C18:2 (GRAS)	Approved: C7-C20; Permitted maximum levels: GMP	Approved: C8:0, C10:0, C12:0, C14:0, C16:0, C18:0, C18:1, C18:2; Permitted maximum levels: GMP	[84-86]
Bases	Sodium bicarbonate	I	144-55-8	Food additive; Permitted maximum levels: Unstandardized confectionery, starch and salt: GMP	Food additive, GRAS	Not approved but preregistered	Approved; Permitted maximum levels: FAO: generally GMP except for infants: 2 g/kg	[87]

	Sodium hydroxide	C, I	1310-73-2	Food additive; Permitted maximum levels: when used together with sodium chloride and calcium oxide: 70 ppm	Food additive, GRAS; Permitted maximum levels: caramel: GMP food starch: 1 %	Not approved	Approved; Permitted maximum levels: FAO: generally GMP except for infants: 2 g/kg	[83]
Biguanides	Chlorhexidine diacetate	A, I, E	56-95-1	Not approved	Not approved	Not approved	Not approved	[66,79]
Ionic liquids	Alkyl methylimidazole chloride	Varies	n.a.	Not approved	Not approved	Not approved	Not approved	[88-90]

* Direct application of the chemicals is currently not approved by the ECHA. Active chlorine and chlorine dioxide released from chlorine containing chemicals such as hydrochloric acid, hypochlorous acid, sodium chlorate, sodium chlorite and tetrachlorodecaoxide complex are being approved. The release of active chlorine from sodium hypochlorite is approved; Substances generally prohibited from use in human food in the U.S. are listed under the Electronic Code of Federal Regulations, Title 21 §189.

C ... Corrosive; A ... Acute toxicity; I ... Irritant; H ... Health Hazard; E ... Environment toxicity; F ... Flammable; G ... compressed Gas

viruses MDPI

References

1. Intralytix. Listshieldtm. http://www.intralytix.com/files/prod/01LP/01LP-Desc.pdf (accessed on 25 January 2019).
2. Intralytix. Ecoshieldtm. Available online: http://www.intralytix.com/files/prod/07EP/07EP-Desc.pdf (accessed on 25 January 2019).
3. Intralytix. Salmofreshtm. Available online: http://www.intralytix.com/files/prod/02SP/02SP-Desc.pdf (accessed on 25 January 2019).
4. Phagelux-Inc. Gras notification: Salmopro®. Available online: http://www.fda.gov/downloads/Food/IngredientsPackagingLabeling/GRAS/NoticeInventory/UCM624100.pdf (accessed on 25 January 2019).
5. Pasternack, G.R.; Sulakelidze, A. *Listeria monocytogenes* bacteriophages and uses thereof. *Intralytix, Inc., Baltimore, MD (US)* **2014**.
6. Pasternack, G.R.; Sulakelidze, A. *Listeria monocytogenes* bacteriophages and uses thereof. *Intralytix, Inc., Baltimore, MD (US)* **2009**.
7. Sulakelidze, A.; Sozhamamnnan, S.; Pasternack, G.R. *Salmonella* bacteriophage and uses thereof. *Intralytix, Inc., Baltimore, MD (US)* **2010**.
8. Pasternack, G.; Sulakelidze, A. *Salmonella* bacteriophage and uses thereof. *Intralytix, Inc., Baltimore, MD (US)* **2014**.
9. Sulakelidze, A.; Pasternack, G.R. *E. Coli* bacteriophage and uses thereof. *Intralytix, Inc., Baltimore, MD (US)* **2009**.
10. Sulakelidze, A.; Pasternack, G.R. *E. Coli* O157:H7 bacteriophage and uses thereof. *Intralytix, Inc., Baltimore, MD (US)* **2009**.
11. Pasternack, G.R.; Sulakelidze, A. *E. Coli* O157:H7 bacteriophage and uses thereof. *Intralytix, Inc., Baltimore, MD (US)* **2009**.
12. Intralytix. Veterinary applications. Available online: http://www.intralytix.com/index.php?page=vet (accessed on 13 February 2019).
13. Miller, R.W.; Skinner, E.J.; Sulakvelidze, A.; Mathis, G.F.; Hofacre, C.L. Bacteriophage therapy for control of necrotic enteritis of broiler chickens experimentally infected with C*lostridium perfringens*. *Avian Dis.* **2010**, *54*, 33-40.
14. Micreos-Food-Safety-BV. Phageguard listex application data sheet cheese. Available online: http://www.phageguard.com/wp-content/uploads/2017/03/PhageGuard-Listex-Application-Data-Sheet-Cheese-Final.pdf (accessed on 25 January 2019).
15. Micreos-Food-Safety-BV. Phageguard listex application data sheet: Salmon. Available online: http://www.phageguard.com/wp-content/uploads/2017/03/PhageGuard-Listex-Aplication-Data-Sheet-Salmon-March-17.pdf (accessed on 25 January 2019).
16. Micreos-Food-Safety-BV. How phages can help reduce listeria risks on salmon. Available online: http://www.phageguard.com/wp-content/uploads/2018/11/PhageGuard-whitepaper-Salmon.pdf (accessed on 25 January 2019).
17. Micreos-Food-Safety-BV. Phageguard listex application data sheet meat. Available online: http://www.phageguard.com/wp-content/uploads/2017/03/PhageGuard-Listex-Aplication-Data-Sheet-RTE-Meat-FINAL.pdf (accessed on 25 January 2019).
18. Micreos-Food-Safety-BV. Phageguard-listex reduces *Listeria* on frozen vegetables. Available online: http://www.phageguard.com/wp-content/uploads/2017/11/PhageGuard-ADS-Frozen-Vegetables.pdf (accessed on 25 January 2019).
19. Micreos-Food-Safety-BV. Phageguard listex application on (food contact) surface areas. Available online: http://www.phageguard.com/wp-content/uploads/2018/09/PhageGuard-Listex-on-food-contact-surface-areas-EU.pdf (accessed on 25 January 2019).
20. Micreos-Food-Safety-BV. Phageguard s reduces *Salmonella* on fresh poultry. Available online: http://www.phageguard.com/wp-content/uploads/2018/03/PhageGuard-ADS-Post-Harvest-Poultry-v82.pdf (accessed on 25 January 2019).
21. Micreos-Food-Safety-BV. Phageguard e reduces *E. Coli* on leafy green vegetables. Available online: http://www.phageguard.com/wp-content/uploads/2018/09/PhageGuard-E-ADS-on-leafy-greens.pdf (accessed on 25 January 2019).

22. Micreos-Food-Safety-BV. Phageguard e reduces e. Coli on beef carcass, parts and trim. Available online: http://www.phageguard.com/wp-content/uploads/2018/09/PhageGuard-E-ADS-beef.pdf (accessed on 25 January 2019).
23. Phagelux-Inc. Gras notification: Salmopro®. Available online: http://www.fda.gov/downloads/food/ingredientspackaginglabeling/gras/noticeinventory/ucm476554.pdf (accessed on 25 January 2019).
24. Passport-Food-Safety-Solutions-Inc. Finalyse® overhead spray system. Available online: http://www.passportfoodsafety.com/assets/pdf/Finalyse Product Insert_2017.03.28_LR.pdf (accessed on 13 February 2019).
25. Passport-Food-Safety-Solutions-Inc. Finalyse®: A novel pre-harvest hide wash. Available online: http://www.passportfoodsafety.com/assets/pdf/Finalyse Detailer_2017.03.28_LR.pdf (accessed on 13 February 2019).
26. OmniLytics. Agriphage. Available online: http://www.agriphage.com/wp-content/uploads/2018/11/67986-1-EPA-Final-Print-Label-AGRIPHAGE-10-18-18.pdf (accessed on 25 January 2019)
27. OmniLytics. Agriphage-cmm. Available online: http://www.agriphage.com/wp-content/uploads/2018/11/67986-6-EPA-Final-Print-Label-AGRIPHAGE-CMM-10-23-18.pdf (accessed on 25 January 2019)
28. OmniLytics. Agriphage-fire blight. Available online: http://www.agriphage.com/wp-content/uploads/2018/11/EPA-Stamped-Label-67986-8-20180927.pdf (accessed on 25 January 2019)
29. OmniLytics. Agriphage-citrus canker. Available online: http://www.agriphage.com/wp-content/uploads/2018/11/EPA-Stamped-Label-67986-9-20180927.pdf (accessed on 25 January 2019)
30. Phagelux-Inc. Available online: Lexia. http://www.phagelux.com/en/single/menu_84.htm?menuid=84 (accessed on 2 April 2019).
31. Sharma, M. Lytic bacteriophages: Potential interventions against enteric bacterial pathogens on produce. *Bacteriophage* **2013**, *3*, e25518-e25518.
32. Enviroinvest-Környezetvédelmi-és-Biotechnológiai-Zrt. Erwiphage plus. Available online: http://www.erwiphage.com (accessed on 13 February 2019).
33. APS-Biocontrol-Ltd. Biolyse®-pb. Available online: http://www.apsbiocontrol.com/products **2019**.
34. FINK-TEC-GmbH. *Escherichia coli*-specific phage preparation. Available online: http://www.fda.gov/downloads/Food/IngredientsPackagingLabeling/GRAS/NoticeInventory/UCM597733.pdf (accessed on 13 February 2019).
35. Proteon-Pharmaceuticals. Bafador®. Available online: http://www.proteonpharma.com/products/bafador-aquaculture/ (accessed on 13 February 2019).
36. Proteon-Pharmaceuticals. Bafasal®. Available online: http://www.proteonpharma.com/products/bafasal-poultry/ (accessed on 13 February 2019).
37. Brimrose-Technology-Corporation. Prevention&treatment-bacteriophage product. Available online: http://www.brimrosetechnology.com/prevention-treatment (accessed on 13 February 2019).
38. Eliava-Pharmacy. Pyo bacteriophage. Available online: http://bacteriophagepharmacy.com/product/pyo-bacteriophage/ (accessed on 13 February 2019).
39. Eliava-Pharmacy. Intesti bacteriophage. Available online: http://bacteriophagepharmacy.com/product/intesti-bacteriophage/ (accessed on 13 February 2019).
40. Eliava-Pharmacy. Ses bacteriophage. **2019**.
41. Eliava-Pharmacy. Enko bacteriophage. Available online: http://bacteriophagepharmacy.com/product/enko-bacteriophage/ (accessed on 13 February 2019).
42. Eliava-Pharmacy. Fersis bacteriophage. Available online: http://bacteriophagepharmacy.com/product/fersis-bacteriophage/ (accessed on 13 February 2019).
43. Eliava-Pharmacy. Staphylococcal bacteriophage. Available online: http://bacteriophagepharmacy.com/product/staphylococcal-bacteriophage/ (accessed on 13 February 2019).
44. Mai, V.; Ukhanova, M.; Visone, L.; Abuladze, T.; Sulakvelidze, A. Bacteriophage administration reduces the concentration of *Listeria monocytogenes* in the gastrointestinal tract and its translocation to spleen and liver in experimentally infected mice. *Int. J. Microbiol.* **2010**, *2010*, 624234.
45. Magnone, J.P.; Marek, P.J.; Sulakvelidze, A.; Senecal, A.G. Additive approach for inactivation of *Escherichia coli* O157:H7, *Salmonella*, and *Shigella spp*. On contaminated fresh fruits and vegetables using bacteriophage cocktail and produce wash. *J. Food Prot.* **2013**, *76*, 1336-1341.

46. Albino, L.A.A.; Rostagno, M.H.; Húngaro, H.M.; Mendonça, R.C.S. Isolation, characterization, and application of bacteriophages for *Salmonella spp*. Biocontrol in pigs. *Foodborne Pathog. Dis.* **2014**, *11*, 602-609.
47. Zhang, H.; Wang, R.; Bao, H. Phage inactivation of foodborne shigella on ready-to-eat spiced chicken. *Poultry Sci.* **2013**, *92*, 211-217.
48. Gras notification of the bacteriophage cocktail: Shigashield tm. Available online: http://www.fda.gov/downloads/food/ingredientspackaginglabeling/gras/noticeinventory/ucm529756.pdf (accessed on 25 January 2019).
49. Boyd, E.F.; Brüssow, H. Common themes among bacteriophage-encoded virulence factors and diversity among the bacteriophages involved. *Trends Microbiol.* **2002**, *10*, 521-529.
50. Micreos-Food-Safety-BV. Salmonelextm notification. Available online: http://www.fda.gov/downloads/Food/IngredientsPackagingLabeling/GRAS/NoticeInventory/ucm505161.pdf (accessed on 25 January 2019).
51. Alagappan, K.M.; Deivasigamani, B.; Somasundaram, S.T.; Kumaran, S. Occurrence of *Vibrio parahaemolyticus* and its specific phages from shrimp ponds in east coast of india. *Curr. Microbiol.* **2010**, *61*, 235-240.
52. Letchumanan, V.; Chan, K.-G.; Pusparajah, P.; Saokaew, S.; Duangjai, A.; Goh, B.-H.; Ab Mutalib, N.-S.; Lee, L.-H. Insights into bacteriophage application in controlling *Vibrio* species. *Front. Microbiol.* **2016**, *7*.
53. Balogh, **B.**; Jone, J.B. Improved efficacy of newly formulated bacteriophages for management of bacterial spot on tomato. *Plant Dis.* **2003**, *87*, 6.
54. Lee, C.-N.; Lin, J.-W.; Weng, S.-F.; Tseng, Y.-H. Genomic characterization of the intron-containing t7-like phage phil7 of *Xanthomonas campestris†*. *Appl. Environ. Microb.* **2009**, *75*, 7828.
55. Yu, J.G.; Lim, J.A.; Song, Y.R.; Heu, S.; Kim, G.H.; Koh, Y.J.; Oh, C.S. Isolation and characterization of bacteriophages against *Pseudomonas syringae* pv. Actinidiae causing bacterial canker disease in kiwifruit. *J. Microbiol. Biotechn.* **2016**, *26*, 385-393.
56. Wittmann, J.; Gartemann, K.-H.; Eichenlaub, R.; Dreiseikelmann, B. Genomic and molecular analysis of phage cmp1 from *Clavibacter michiganensis* subspecies *michiganensis*. *Bacteriophage* **2011**, *1*, 6-14.
57. Gill, J.J.; Svircev, A.M.; Smith, R.; Castle, A.J. Bacteriophages of *Erwinia amylovora*. *Appl. Environ. Microb.* **2003**, *69*, 2133.
58. Born, Y.; Fieseler, L.; Marazzi, J.; Lurz, R.; Duffy, B.; Loessner, M.J. Novel virulent and broad-host-range *Erwinia amylovora* bacteriophages reveal a high degree of mosaicism and a relationship to enterobacteriaceae phages. *Appl. Environ. Microb.* **2011**, *77*, 5945.
59. Ahmad, A.A.; Ogawa, M.; Kawasaki, T.; Fujie, M.; Yamada, T. Characterization of bacteriophages cp1 and cp2, the strain-typing agents for *Xanthomonas axonopodis* pv. Citri. *Appl. Environ. Microb.* **2014**, *80*, 77.
60. Meczker, K.; Domotor, D.; Vass, J.; Rakhely, G.; Schneider, G.; Kovacs, T. The genome of the *Erwinia amylovora* phage phieah1 reveals greater diversity and broadens the applicability of phages for the treatment of fire blight. *FEMS Microbiol. Lett.* **2014**, *350*, 25-27.
61. Adriaenssens, E.M.; Van Vaerenbergh, J.; Vandenheuvel, D.; Dunon, V.; Ceyssens, P.-J.; De Proft, M.; Kropinski, A.M.; Noben, J.-P.; Maes, M.; Lavigne, R. T4-related bacteriophage limestone isolates for the control of soft rot on potato caused by 'dickeya solani'. *PLOS ONE* **2012**, *7*, e33227.
62. O'Sullivan, L.; Bolton, D.; McAuliffe, O.; Coffey, A. Bacteriophages in food applications: From foe to friend. *Annu. Rev. Food Sci. Technol.* **2019**.
63. Pasternack, G.; Sulakelidze, A. Novel shigella bacteriophages and uses thereof. *Intralytix, Inc., Baltimore, MD (US)* **2016**.
64. D'Souza, D.H.; Su, X. Efficacy of chemical treatments against murine norovirus, feline calicivirus, and ms2 bacteriophage. *Foodborne Pathog. Dis.* **2010**, *7*, 319-326.
65. Kobayashi, H.; Tsuzuki, M.; Koshimizu, K.; Toyama, H.; Yoshihara, N.; Shikata, T.; Abe, K.; Mizuno, K.; Otomo, N.; Oda, T. Susceptibility of hepatitis b virus to disinfectants or heat. *J. Clin. Microbiol.* **1984**, *20*, 214-216.
66. Maillard, J.Y.; Beggs, T.S.; Day, M.J.; Hudson, R.A.; Russell, A.D. Effect of biocides on ms2 and k coliphages. *Appl. Environ. Microb.* **1994**, *60*, 2205-2206.
67. Cook, A.M.; Brown, W.R.L. Inactivation of a bacteriophage by chemical antibacterial agents. *J. Pharm. Pharmacol.* **1964**, *16*, 611-617.

68. Smith, H.W.; Huggins, M.B.; Shaw, K.M. Factors influencing the survival and multiplication of bacteriophages in calves and in their environment. *J. Gen. Microbiol.* **1987**, *133*, 1127-1135.
69. Brown, B.L.; Fuerst, R. Ethylene oxide sterilization of tissue culture media. *Science (New York, N.Y.)* **1963**, *142*, 1654-1655.
70. Thurston-Enriquez, J.A.; Haas, C.N.; Jacangelo, J.; Gerba, C.P. Chlorine inactivation of adenovirus type 40 and feline calicivirus. *Appl. Environ. Microb.* **2003**, *69*, 3979-3985.
71. Zyara, A.M.; Torvinen, E.; Veijalainen, A.M.; Heinonen-Tanski, H. The effect of chlorine and combined chlorine/uv treatment on coliphages in drinking water disinfection. *J. Water Health* **2016**, *14*, 640-649.
72. Campagna, C.; Villion, M.; Labrie, S.J.; Duchaine, C.; Moineau, S. Inactivation of dairy bacteriophages by commercial sanitizers and disinfectants. *Int. J. Food Microbiol.* **2014**, *171*, 41-47.
73. Grunert, A.; Frohnert, A.; Selinka, H.C.; Szewzyk, R. A new approach to testing the efficacy of drinking water disinfectants. *Int. J. Hyg. Environ. Health* **2018**, *221*, 1124-1132.
74. Binetti, A.G.; Reinheimer, J.A. Thermal and chemical inactivation of indigenous *Streptococcus thermophilus* bacteriophages isolated from argentinian dairy plants. *J. Food Prot.* **2000**, *63*, 509-515.
75. Ebrecht, A.C.; Guglielmotti, D.M.; Tremmel, G.; Reinheimer, J.A.; Suarez, V.B. Temperate and virulent *Lactobacillus delbrueckii* bacteriophages: Comparison of their thermal and chemical resistance. *Food Microbiol.* **2010**, *27*, 515-520.
76. Briggiler Marco, M.; De Antoni, G.L.; Reinheimer, J.A.; Quiberoni, A. Thermal, chemical, and photocatalytic inactivation of *Lactobacillus plantarum* bacteriophages. *J. Food Prot.* **2009**, *72*, 1012-1019.
77. Dunkin, N.; Weng, S.; Schwab, K.J.; McQuarrie, J.; Bell, K.; Jacangelo, J.G. Comparative inactivation of murine norovirus and ms2 bacteriophage by peracetic acid and monochloramine in municipal secondary wastewater effluent. *Environ. Sci. Technol.* **2017**, *51*, 2972-2981.
78. Hayes, S.; Murphy, J.; Mahony, J.; Lugli, G.A.; Ventura, M.; Noben, J.-P.; Franz, C.M.A.P.; Neve, H.; Nauta, A.; Van Sinderen, D. Biocidal inactivation of *Lactococcus lactis* bacteriophages: Efficacy and targets of commonly used sanitizers. *Front. Microbiol.* **2017**, *8*, 107-107.
79. Agun, S.; Fernandez, L.; Gonzalez-Menendez, E.; Martinez, B.; Rodriguez, A.; Garcia, P. Study of the interactions between bacteriophage phiipla-rodi and four chemical disinfectants for the elimination of *Staphylococcus aureus* contamination. *Viruses* **2018**, *10*.
80. Morin, T.; Martin, H.; Soumet, C.; Fresnel, R.; Lamaudiere, S.; Le Sauvage, A.L.; Deleurme, K.; Maris, P. Comparison of the virucidal efficacy of peracetic acid, potassium monopersulphate and sodium hypochlorite on bacteriophages p001 and ms2. *J. Appl. Microbiol.* **2015**, *119*, 655-665.
81. Tomat, D.; Balagué, C.; Aquili, V.; Verdini, R.; Quiberoni, A. Resistance of phages lytic to pathogenic *Escherichia coli* to sanitisers used by the food industry and in home settings. *Food Sci. Technol. Int.* **2018**, *53*, 533-540.
82. Mercanti, D.J.; Guglielmotti, D.M.; Patrignani, F.; Reinheimer, J.A.; Quiberoni, A. Resistance of two temperate *Lactobacillus paracasei* bacteriophages to high pressure homogenization, thermal treatments and chemical biocides of industrial application. *Food Microbiol.* **2012**, *29*, 99-104.
83. Branston, S.D.; Stanley, E.C.; Ward, J.M.; Keshavarz-Moore, E. Determination of the survival of bacteriophage m13 from chemical and physical challenges to assist in its sustainable bioprocessing. *Biotechnol. Bioproc. E.* **2013**, *18*, 560-566.
84. Sands, J.A. Inactivation and inhibition of replication of the enveloped bacteriophage phi6 by fatty acids. *Antimicrob. Agents Chemother.* **1977**, *12*, 523-528.
85. Hirotani, H.; Ohigashi, H.; Kobayashi, M.; Koshimizu, K.; Takahashi, E. Inactivation of t5 phage by cis - vaccenic acid, an antivirus substance from *Rhodopseudomonas capsulata*, and by unsaturated fatty acids and related alcohols. *FEMS Microbiol. Lett.* **1991**, *77*, 13-18.
86. Reinhardt, A.; Cadden, S.; Sands, J.A. Inhibitory effect of fatty acids on the entry of the lipid-containing bacteriophage pr4 into *Escherichia coli*. *J. Virol.* **1978**, *25*, 479-485.
87. Allwood, P.B.; Malik, Y.S.; Hedberg, C.W.; Goyal, S.M. Effect of temperature and sanitizers on the survival of feline calicivirus, *Escherichia coli*, and f-specific coliphage ms2 on leafy salad vegetables. *J. Food Prot.* **2004**, *67*, 1451-1456.
88. Sommer, J.; Fister, S.; Gundolf, T.; Bromberger, B.; Mester, P.-J.; Witte, A.; Kalb, R.; Rossmanith, P. Virucidal or not virucidal? That is the question—predictability of ionic liquid's virucidal potential in biological test systems. *Int. J. Mol. Sci.* **2018**, *19*, 790.
89. Fister, S.; Mester, P.; Sommer, J.; Witte, A.K.; Kalb, R.; Wagner, M.; Rossmanith, P. Virucidal influence of ionic liquids on phages p100 and ms2. *Front. Microbiol.* **2017**, *8*.

90. Byrne, N.; Rodoni, B.; Constable, F.; Varghese, S.; Davis, J.H. Enhanced stabilization of the tobacco mosaic virus using protic ionic liquids. *Phys. Chem. Chem. Phys.* **2012**, *14*, 10119-10121.

© 2019 by the authors. Submitted for possible open access publication under the terms and conditions of the Creative Commons Attribution (CC BY) license (http://creativecommons.org/licenses/by/4.0/).
